# Cotargeting a MYC/eIF4A-survival axis improves the efficacy of KRAS inhibitors in lung cancer

**DOI:** 10.1172/JCI167651

**Published:** 2023-08-15

**Authors:** Francesca Nardi, Naiara Perurena, Amy E. Schade, Ze-Hua Li, Kenneth Ngo, Elena V. Ivanova, Aisha Saldanha, Chendi Li, Prafulla C. Gokhale, Aaron N. Hata, David A. Barbie, Cloud P. Paweletz, Pasi A. Jänne, Karen Cichowski

**Affiliations:** 1 Genetics Division and; 2 Department of Medicine, Brigham and Women’s Hospital, Boston, Massachusetts, USA.; 3 Harvard Medical School, Boston, Massachusetts, USA.; 4 Ludwig Center at Harvard, Boston, Massachusetts, USA.; 5 Department of Medical Oncology and; 6 Belfer Center for Applied Cancer Science, Dana-Farber Cancer Institute, Boston, Massachusetts, USA.; 7 Massachusetts General Hospital Cancer Center, Charlestown, Massachusetts, USA.; 8 Depertment of Medicine, Massachusetts General Hospital, and Harvard Medical School, Boston, Massachusetts, USA.; 9 Experimental Therapeutics Core and; 10 Lowe Center for Thoracic Oncology, Dana-Farber Cancer Institute, Boston, Massachusetts, USA.

**Keywords:** Oncology, Drug therapy, Lung cancer, Signal transduction

## Abstract

Despite the success of KRAS G12C inhibitors in non–small cell lung cancer (NSCLC), more effective treatments are needed. One preclinical strategy has been to cotarget RAS and mTOR pathways; however, toxicity due to broad mTOR inhibition has limited its utility. Therefore, we sought to develop a more refined means of targeting cap-dependent translation and identifying the most therapeutically important eukaryotic initiation factor 4F complex–translated (eIF4F-translated) targets. Here, we show that an eIF4A inhibitor, which targets a component of eIF4F, dramatically enhances the effects of KRAS G12C inhibitors in NSCLCs and together these agents induce potent tumor regression in vivo. By screening a broad panel of eIF4F targets, we show that this cooperativity is driven by effects on BCL-2 family proteins. Moreover, because multiple BCL-2 family members are concomitantly suppressed, these agents are broadly efficacious in NSCLCs, irrespective of their dependency on MCL1, BCL-xL, or BCL-2, which is known to be heterogeneous. Finally, we show that MYC overexpression confers sensitivity to this combination because it creates a dependency on eIF4A for BCL-2 family protein expression. Together, these studies identify a promising therapeutic strategy for *KRAS*-mutant NSCLCs, demonstrate that BCL-2 proteins are the key mediators of the therapeutic response in this tumor type, and uncover a predictive biomarker of sensitivity.

## Graphical abstract

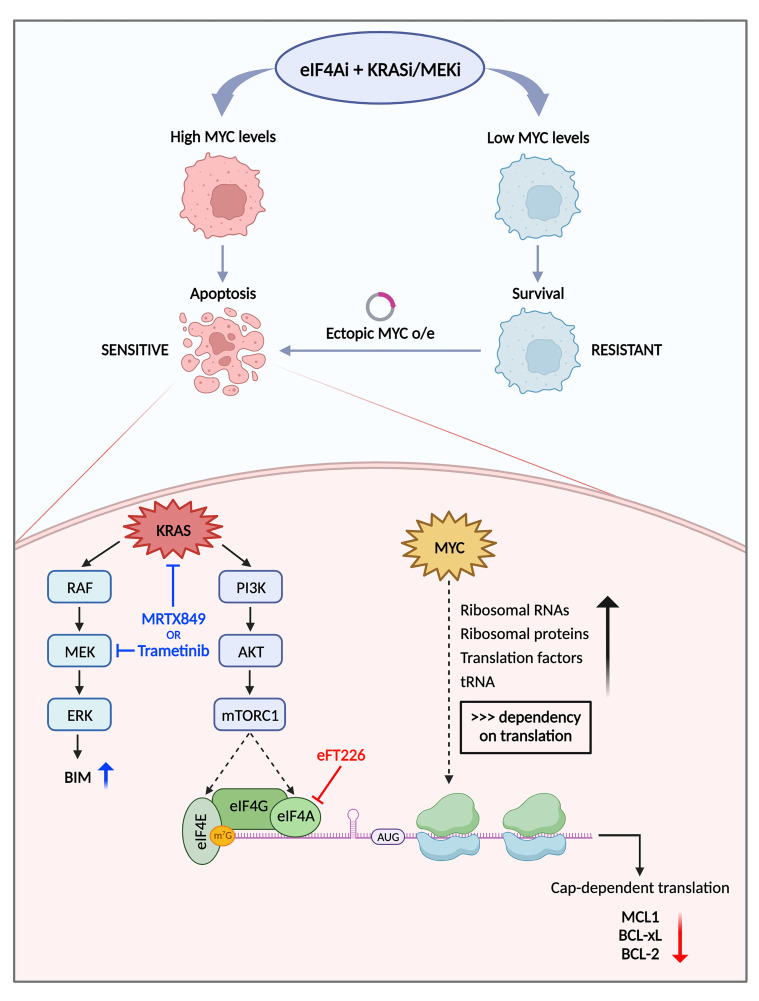

## Introduction

While activating mutations in the *KRAS* oncogene occur in nearly one-third of non–small cell lung cancers (NSCLCs), effective therapeutic options for these malignancies remain limited (1). Fortunately, covalent inhibitors of KRAS G12C, the most frequent *KRAS* mutation in NSCLCs (~40%), have been developed and are showing promise in the clinic (2, 3). However, fewer than half of patients respond to these agents and responses are temporary (4, 5). In addition, KRAS G12C inhibitors are not relevant for the remaining approximately 60% of *KRAS*-mutant NSCLCs that harbor other mutant *KRAS* alleles (3, 6).

Several strategies are being employed to improve the efficacy of KRAS G12C inhibitors. Many of these approaches are designed to enhance the suppression of RAS signaling and include the cosuppression of either the RAS effector MEK, receptor tyrosine kinases (RTKs), or the SHP2 phosphatase, which potentiates RAS signaling downstream of RTKs (7–9). Combinations of KRAS G12C inhibitors and either MEK or RTK inhibitors have already been evaluated in clinical trials; however, to date, response rates do not appear to be appreciably superior to those with single-agent KRAS G12C inhibition (CodeBreak 101 Trial; NCT04185883). Regardless, new therapeutic strategies are urgently needed to improve the outcome of patients with *KRAS G12C*–mutant cancers.

Dysregulation of mRNA translation is a critical oncogenic event in human cancer, and tumors frequently reprogram translational control mechanisms to support growth and survival and to rapidly adapt to microenvironmental changes (10). Cap-dependent translation, in particular, is commonly hyperactivated in tumors through genetic alterations in components of the eukaryotic initiation factor 4F complex (eIF4F) or due to aberrant activation of oncogenic pathways, including RAS/ERK, PI3K/mTOR, and MYC, which all converge on this complex (10–12). Notably, eIF4F does not control the translation of all mRNAs, but rather a subset that possess specific secondary structures in their 5′ UTRs (13). However, many of these mRNAs encode proteins that promote proliferation, survival, metastasis, and immune evasion (11, 14–16). Therefore, aberrant activation of the eIF4F complex selectively enhances the expression of important protumorigenic genes.

A key component of the eIF4F complex is the RNA helicase eIF4A, which is required to unwind the secondary structures of cap-dependent transcripts (10, 17). Because many oncogenic transcripts contain long and highly structured 5′ UTRs, they require eIF4A activity for their translation, suggesting that cancer cells may be more dependent on the activity of this helicase than normal cells (13). Therefore, we hypothesized that inhibition of eIF4A might improve the efficacy of KRAS inhibitors by selectively suppressing the enhanced translation of specific protumorigenic mRNAs. We further reasoned that cotargeting a downstream node (the eIF4F complex) that serves as a convergence point for multiple oncogenic signals might also mitigate the need for deeper RAS pathway suppression and could ultimately be more selective for cancer cells. Fortunately, a potent and selective small-molecule inhibitor of eIF4A, eFT226, has recently been developed and is currently being evaluated in clinical trials (18). Interim results show favorable tolerability, even when combined with other agents, and signs of clinical activity (19).

In this study, we show that KRAS G12C and eIF4A inhibitors dramatically cooperate and potently kill NSCLCs in vitro and in vivo. By screening a broad panel of direct eIF4F translational targets and performing functional studies, we identify the critical mediators of this response in NSCLCs. We also demonstrate that eIF4A inhibition similarly cooperates with MEK inhibitors, offering a potential therapeutic strategy for tumors that harbor non-G12C mutant *KRAS* alleles. Finally, we show that MYC is a driver and biomarker of the observed effects because it creates a dependency on eIF4A/F for the expression of prosurvival proteins. Together, these studies identify two promising therapeutic strategies for *KRAS*-mutant NSCLCs and reveal important insights into the biological consequences of targeting the eIF4F complex in lung cancer.

## Results

### eIF4A inhibitors dramatically sensitize NSCLCs to KRAS G12C inhibitors.

**Figure 1:**
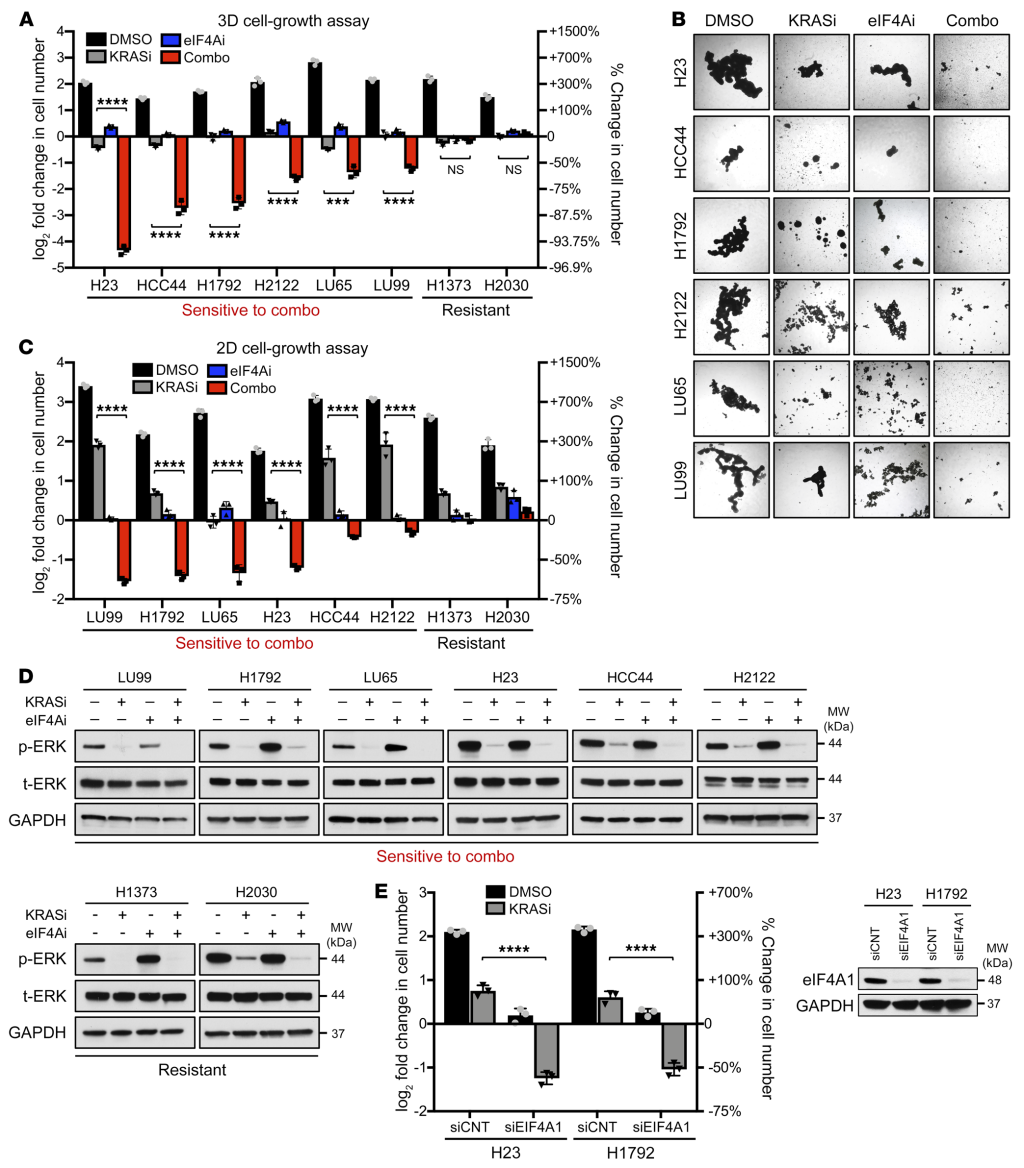
eIF4A inhibitors dramatically sensitize NSCLCs to KRAS G12C inhibitors. (**A**) Bar graph depicting the fold change in cell numbers after 72 hours (versus day 0) using a panel of *KRAS G12C*–mutant NSCLC cells grown in 3D conditions. Cells were treated with vehicle (DMSO), 25 nM eFT226 (eIF4Ai), 100 nM MRTX849 (KRASi), or both agents together (Combo). Data are represented as mean ± SD. *n* = 3. ***P < 0.001; ****P < 0.0001, 1-way ANOVA and Tukey’s post hoc test. (**B**) Representative photographs of sensitive *KRAS G12C*–mutant NSCLC spheroids after 72 hours of indicated drug treatments. Images were obtained using the 2X Bright Field channel. Original magnification, ×2. (**C**) Bar graph depicting fold change in cell numbers after 72 hours (versus day 0) in the same panel of cell lines grown in 2D tissue-culture conditions with 2% serum. Cells were treated with vehicle (DMSO), 25 nM eFT226 (eIF4Ai), 100 nM MRTX849 (KRASi), or both agents together (Combo). Data are represented as means ± SD. *n* = 3. ****P < 0.0001, 1-way ANOVA and Tukey’s post hoc test). (**D**) Immunoblots showing suppression of phospho-ERK by MRTX849 (KRASi, 100 nM) and/or eFT226 (eIF4Ai, 25 nM) after 24 hours of treatment. (**E**) Bar graphs depicting fold change in cell number of specified cell lines transfected with either siCNT or siEIF4A1 and treated with vehicle (DMSO) or 100 nM MRTX849 (KRASi) for 72 hours in 2D/low-serum conditions. Data are represented as means ± SD. *n* = 3. ****P < 0.0001, 1-way ANOVA and Tukey’s post hoc test. Immunoblots confirm suppression of eIF4A1 by siRNAs.

To determine whether eIF4A suppression could enhance the effects of KRAS G12C inhibition, we evaluated the eIF4A inhibitor eFT226 and the KRAS G12C inhibitor MRTX849 in a panel of NSCLC cell lines harboring the *KRAS G12C*–mutant allele. Because KRAS G12C inhibitors have been shown to be more effective in 3D cell-growth assays, the effects of these agents were first assessed under conditions that promote spheroid formation, as previously described (3, 20) (Figure 1A). MRTX849 (KRASi, 100 nM) alone triggered cytostatic or modestly cytotoxic effects in all cell lines examined, whereas eFT226 alone (eIF4Ai, 25 nM) exclusively conferred cytostatic responses (Figure 1, A and B). However, eIF4A inhibitors potently sensitized 75% of the cell lines to KRAS G12C inhibitors, resulting in the loss of approximately 60%–90% of cells within just 3 days, as quantified in Figure 1A, with images shown in Figure 1B. Cell lines that exhibited an enhanced response to this combination, as compared with KRAS G12C inhibitors alone, are herein referred to as “sensitive,” whereas “resistant” cells are defined as those with no enhanced cytotoxic response.

We also investigated the effects of these agents in 2D cultures in the presence of low levels of serum (2%), conditions that may recapitulate the limited nutrients/growth factors available to tumor cells in vivo. Notably, all cell lines identified as sensitive in 3D were also sensitive to this drug combination in the modified 2D assay, albeit with slightly different relative sensitivities (Figure 1C), suggesting that this assay may provide an alternative, more tractable method for evaluating KRAS G12C inhibitor–based combinations. Regardless, in both sensitive and resistant cells, MRTX849 effectively inhibited RAS signaling, as demonstrated by the suppression of phosphorylated ERK (phospho-ERK) (Figure 1D). It should be noted that eFT226 did not appreciably enhance the suppression of phospho-ERK in these cell lines, suggesting that it was not functioning by promoting a deeper suppression of RAS/ERK signaling (Figure 1D). Additional upstream and downstream components of the RAS pathway will be further discussed below. Importantly, genetic ablation of eIF4A recapitulated the effects of eFT226 and cooperatively killed cells when combined with KRAS G12C inhibitors (Figure 1E).

### eIF4A inhibitors similarly enhance the effects of MEK inhibitors.

**Figure 2:**
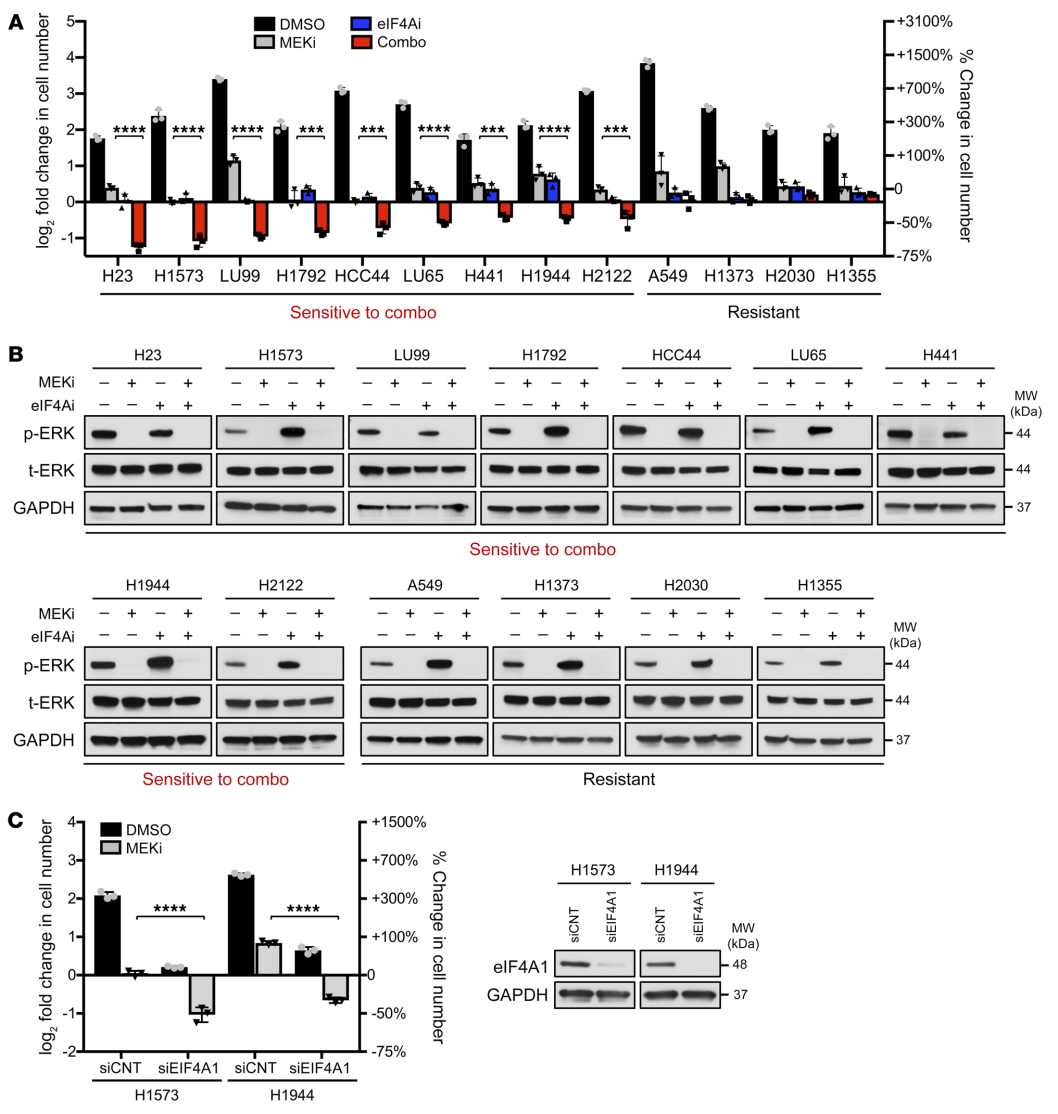
eIF4A inhibitors similarly enhance the effects of MEK inhibitors. (**A**) Bar graph depicting fold change in cell numbers in NSCLC cell lines harboring various mutations in *KRAS* treated with vehicle (DMSO), 25 nM eFT226 (eIF4Ai), 50 nM trametinib (MEKi), or both agents together (Combo) for 72 hours in 2D/low-serum conditions. Data are represented as means ± SD. *n* = 3. ***P < 0.001; ****P < 0.0001, 1-way ANOVA and Tukey’s post hoc test. H23 (*KRAS G12C*), H1573 (*KRAS G12A*), LU99 (*KRAS G12C*), H1792 (*KRAS G12C*), HCC44 (*KRAS G12C*), LU65 (*KRAS G12C*), H441 (*KRAS G12V*), H1944 (*KRAS G13D*), H2122 (*KRAS G12C*), A549 (*KRAS G12S*), H1373 (*KRAS G12C*), H2030 (*KRAS G12C*), H1355 (*KRAS G13C*). (**B**) Immunoblots for each cell line showing suppression of phospho-ERK by trametinib (MEKi) after 24 hours. (**C**) Bar graphs depicting fold change in cell numbers of specified cell lines transfected with either siCNT or siEIF4A1 and treated with vehicle (DMSO) or 50 nM trametinib (MEKi) for 72 hours in 2D/low-serum conditions. Data are represented as means ± SD. *n* = 3. ****P < 0.0001, 1-way ANOVA and Tukey’s post hoc test. Immunoblots confirm suppression of eIF4A1 by siRNAs.

Because KRAS G12C inhibitors only target a single *KRAS* mutation, we used a l«arger panel of cell lines with various activating *KRAS* mutations to determine whether eIF4A inhibition could also enhance the effects of MEK inhibitors. While the MEK inhibitor trametinib broadly exerted cytostatic effects, the addition of eFT226 resulted in a potent cytotoxic response in 9 out of 13 cell lines (Figure 2A). Importantly, trametinib effectively suppressed the RAS pathway in both sensitive and resistant lines, as shown by the suppression of phospho-ERK (Figure 2B). Finally, genetic ablation of eIF4A similarly cooperated with MEK inhibitors to kill tumor cells (Figure 2C). Taken together, these results demonstrate that eIF4A is an important therapeutic target in *KRAS*-mutant NSCLC cells and that suppression of this eIF4F component dramatically sensitizes lung cancer cells to both KRAS G12C and MEK inhibitors.

### eIF4A inhibitors synergize with KRAS G12C inhibitors in NSCLCs, trigger apoptosis, and promote durable responses in vitro and in vivo.

**Figure 3:**
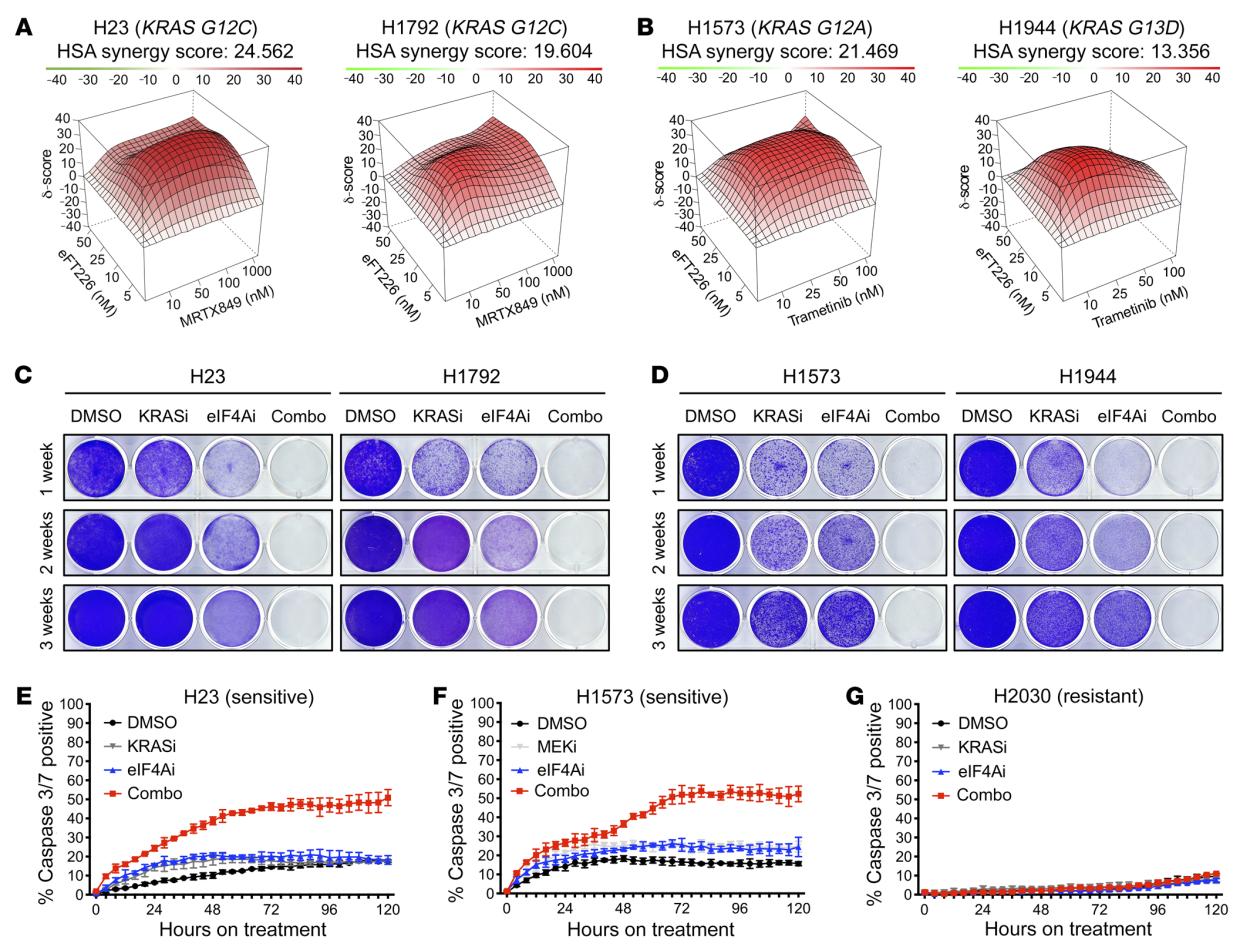
eIF4A inhibitors synergize with KRAS G12C or MEK inhibitors and trigger apoptosis in NSCLCs. (**A** and **B**) Synergy plots depicting the effects of indicated drug combinations using the HSA model. (**C** and **D**) Long-term cell proliferation assay of *KRAS*-mutant NSCLC cells treated with vehicle (DMSO), 25 nM eFT226 (eIF4Ai), 100 nM MRTX849 (KRASi), 50 nM trametinib (MEKi), or drug combinations (Combo) up to 3 weeks. (**E**–**G**) Incucyte live-cell imaging data depicting cleaved caspase-3/7 activity in sensitive H23 and H1573 and resistant H2030 cell lines in response to vehicle (DMSO), 25 nM eFT226 (eIF4Ai), 100 nM MRTX849 (KRASi), 50 nM trametinib (MEKi), or drug combinations (Combo).

Importantly, we found that eFT226 potently synergized with MRTX849 and with trametinib in multiple cell lines, as determined by the highest single agent (HSA) synergy model (Figure 3, A and B, and Supplemental Figure 1A; supplemental material available online with this article; https://doi.org/10.1172/JCI167651DS1). To assess the durability of these responses, *KRAS G12C*–mutant cells were treated with eFT226 and MRTX849 for 3 weeks (Figure 3C and Supplemental Figure 1B). Cells harboring other *KRAS*-mutant alleles were similarly exposed to eFT226 and trametinib over the same time period (Figure 3D). Notably, both combinations effectively killed multiple *KRAS*-mutant NSCLC lines and there were no signs of regrowth throughout the duration of this study (Figure 3, C and D, and Supplemental Figure 1B).

To investigate whether sensitive cells were dying via apoptosis, caspase-3/7 activity was measured using live-cell imaging analysis. MRTX849 alone induced low levels of apoptosis, and notably, eFT226 did so as well (Figure 3E). However, when combined, these agents cooperatively triggered apoptosis in approximately 50% of cells after only 48 to 72 hours (Figure 3E). The combination of eFT226 and trametinib exerted similar effects (Figure 3F). In contrast, little to no apoptosis in response to this drug combination was observed in resistant cells (Figure 3G).

**Figure 4:**
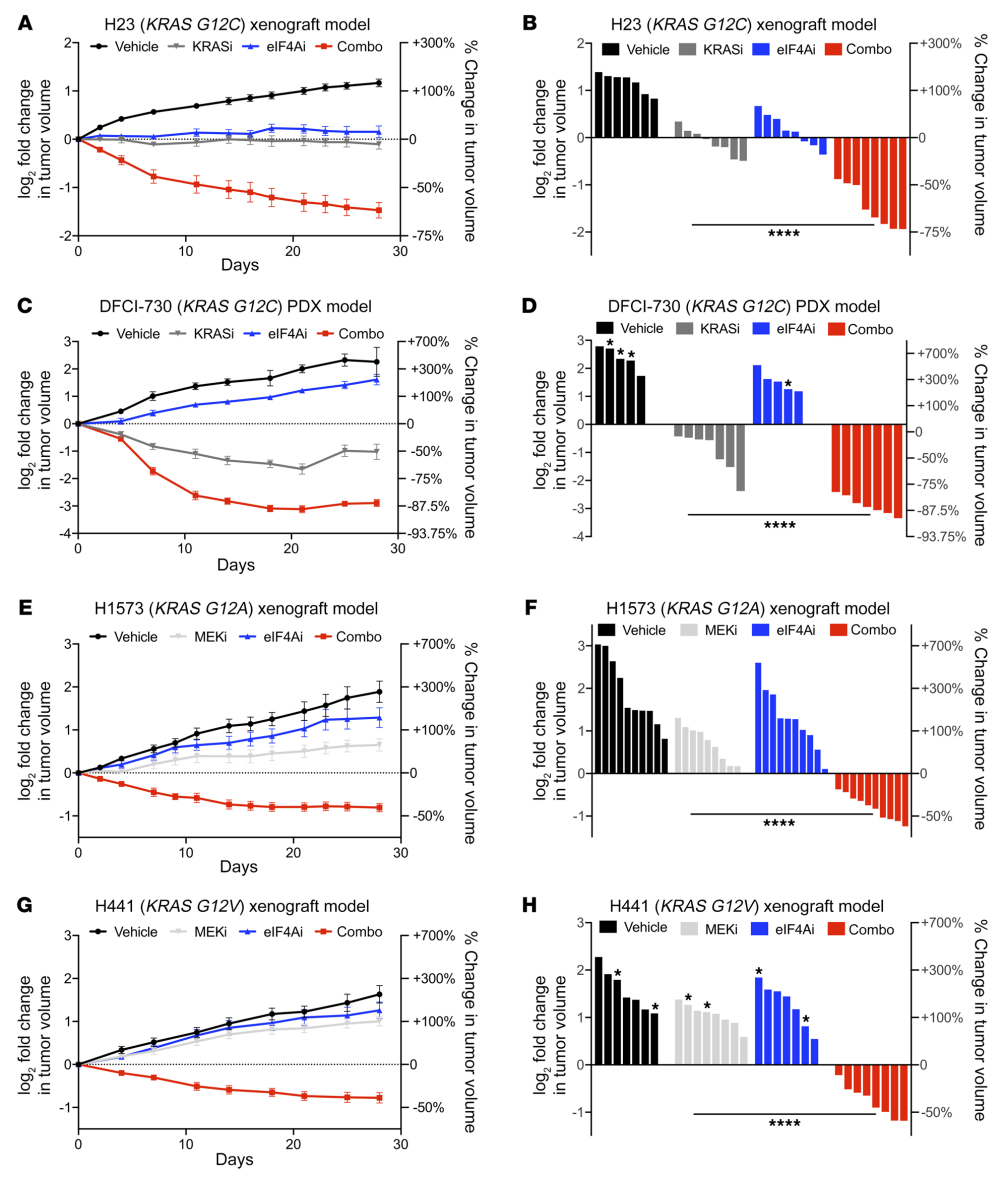
Combined eIF4A and RAS/ERK pathway inhibitors promote potent and durable responses in vivo. Graphs depicting the fold change in tumor volume of (**A** and **B**) H23-derived xenograft models and (**C** and **D**) DFCI-730 PDX models treated for 28 days with vehicle, 100 mg/kg QD MRTX849 (KRASi), 0.5 mg/kg Q4D eFT226 (eIF4Ai), or the 2 agents together (Combo). Data are represented as means ± SEM. *n* = 7–8 tumors per condition. (**B** and **D**) Waterfall plots depicting fold change of each tumor within the 4 treatment arms after 28 days of treatment (versus day 0). ****P < 0.0001, 1-way ANOVA and Tukey’s post hoc test. Single asterisks indicate maximum tumor volume. These mice reached end point at days 21 and 25. Graphs depicting the fold change in tumor volume of (**E** and **F**) H1573-derived xenograft models and (**G** and **H**) H441-derived xenograft models treated for 28 days with vehicle, 0.6 mg/kg QD trametinib (MEKi), 0.5 mg/kg Q4D eFT226 (eIF4Ai), or the 2 agents together (Combo). Data are represented as means ± SEM. *n* = 7–10 tumors per condition. (**F** and **H**) Waterfall plots depicting fold change of each tumor within the 4 treatment arms after 28 days of treatment (versus day 0). ****P < 0.0001, 1-way ANOVA and Tukey’s post hoc test. Single asterisks indicate maximum tumor volume. These mice reached end point at days 18 and 21.

Finally, to determine whether cosuppression of eIF4A and the RAS/ERK pathway could exert similar cooperative effects in vivo, we evaluated the efficacy of eIF4A inhibitors combined with either KRAS G12C or MEK inhibitors in multiple models. First, *KRAS G12C*–mutant xenografts were generated using the H23-sensitive cell line. Mice with established tumors were treated with vehicle, MRTX849 (100 mg/kg once a day [QD]), eFT226 (0.5 mg/kg once every 4 days [Q4D]), or both agents together for 4 weeks. As shown in Figure 4, A and B, both single agents exerted cytostatic or modestly cytotoxic effects; however, when combined, tumors regressed 50%–75% over 28 days. Importantly, regression was durable, and tumors continued to regress throughout the study (Figure 4, A and B). Similarly to what occurred in the xenograft model, combined KRAS G12C and eIF4A inhibitors also caused potent and durable tumor regression in a *KRAS G12C*–mutant patient-derived xenograft (PDX) model (DFCI-730), with tumors regressing up to 85%–90% in response to the drug combination (Figure 4, C and D). Two additional xenograft models were generated using either H1573 cells, which harbor the *KRAS G12A*-mutant allele, or H441 cells, which harbor the *KRAS G12V*-mutant allele. For these studies, mice were treated with vehicle, trametinib (0.6 mg/kg QD), eFT226 (0.5 mg/kg Q4D), or both agents together. Trametinib alone had little effect in these models, consistent with observed inefficacy of MEK inhibitors in NSCLC clinical trials (21, 22). However, the addition of eFT226 promoted susta0ined tumor regression in both models (Figure 4, E–H). As expected, eFT226 did not improve the efficacy of the KRAS G12C inhibitor in the resistant H2030-derived xenograft model, consistent with our in vitro findings (Supplemental Figure 2). Together, these studies demonstrate that eIF4A inhibition substantially enhances the efficacy of RAS/ERK pathway inhibitors both in vitro and in vivo, causing durable regression even in tumors that are minimally sensitive to KRAS G12C or MEK inhibitors.

### eIF4A inhibitors suppress the expression of prosurvival BCL-2 family proteins, cyclin D1, and CDK4 in sensitive NSCLCs.

**Figure 5:**
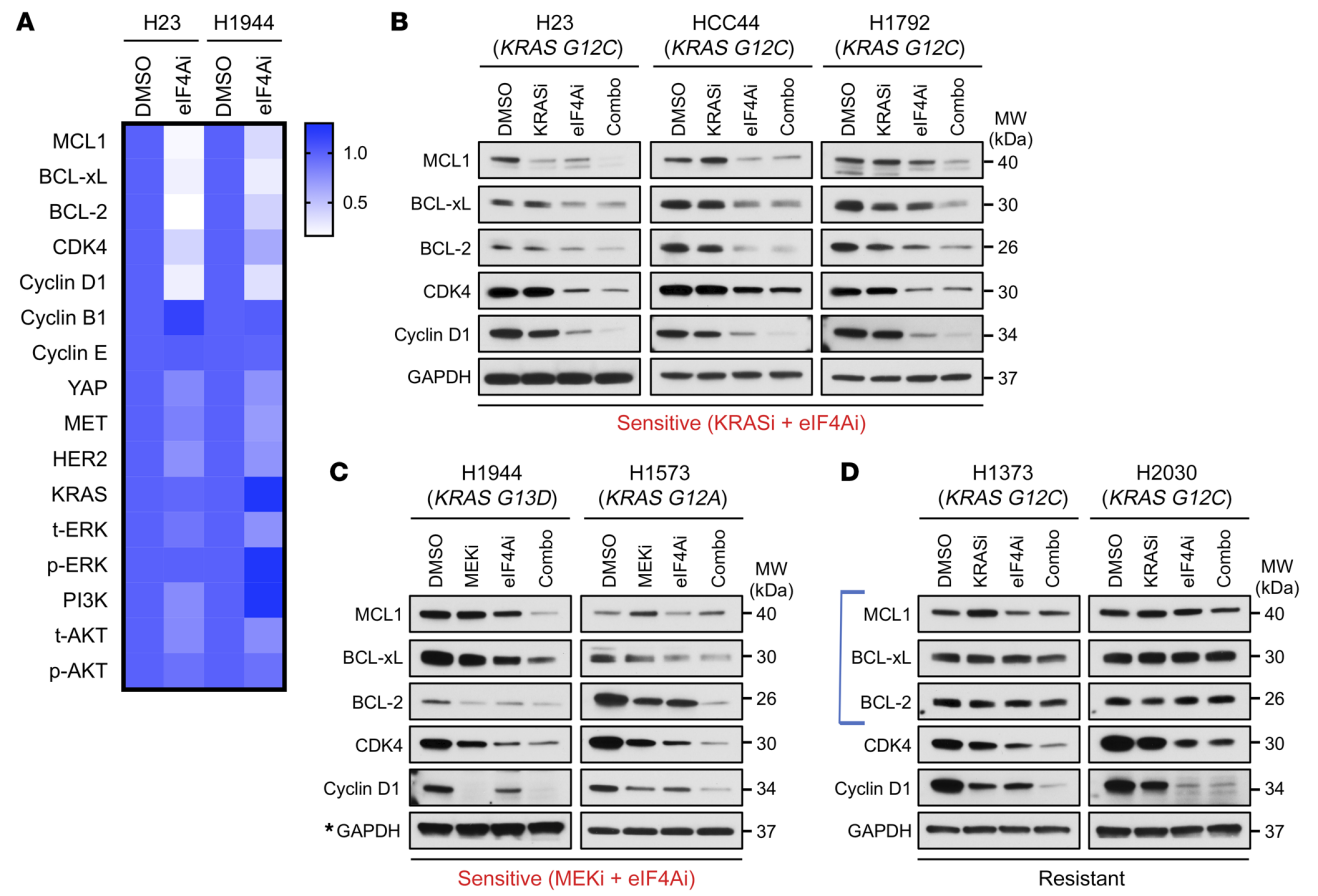
eIF4A and RAS pathway inhibitors cooperatively suppress the expression of prosurvival BCL-2 family proteins, cyclin D1, and CDK4. (**A**) Heatmap depicting protein expression of established eIF4A-regulated targets in H23- and H1944-sensitive lines after 24 hours of treatment with vehicle (DMSO) or eFT226 (eIF4Ai). Protein expression was normalized by GAPDH. Raw data from Western blots are shown in Supplemental Figure 3. (**B** and **C**) Immunoblots of MCL1, BCL-xL, BCL-2, CDK4, and cyclin D1 protein levels in sensitive cell lines after 24 hours of specified treatments. *For H1944, the loading control is the same as shown in Figure 2B because protein expression was tested using the same membrane. (**D**) Immunoblots of the same targets in resistant cell lines after 24 hours of specified treatments.

Numerous direct eIF4A translational targets have been identified by ribosome- and polysome-profiling studies and include proteins involved in cell-cycle progression (e.g., cyclin D1, -B1, -E, CDK4), survival (e.g., MCL1, BCL-xL, BCL-2), transcription (e.g., YAP), metastasis (e.g., MMPs), angiogenesis (e.g., VEGF), immune recognition (e.g., PD-L1), and cell signaling (e.g., RAS, ERK, AKT, PI3K, MET, HER2) (14–16, 23–25). However, the translational control of these targets by eIF4A appears to be very tumor-type specific (11, 15). To identify the critical eIF4A-regulated targets that, when suppressed, sensitize NSCLCs to KRAS G12C or MEK inhibitors, we performed a screen to interrogate 14 established, direct eIF4A translational targets and downstream signaling pathways in response to eFT226 (eIF4Ai) in 2 different sensitive NSCLC lines, eliminating the few targets that would not be expected to exert cell-autonomous effects (e.g., MMPs, PD-L1). Notably, eIF4A inhibition potently suppressed the expression of prosurvival BCL-2 family proteins (MCL1, BCL-xL, and BCL-2) and the cell-cycle proteins cyclin D1 and CDK4 in both sensitive cell lines as compared with untreated cells (Figure 5A, heatmap of protein expression; Supplemental Figure 3, Western blots). In contrast, eIF4A suppression had little to no effect on the expression of other targets, including cyclin B1, cyclin E, YAP, and oncogenic signaling molecules, such as RAS, PI3K, AKT, ERK, MET, and HER2, suggesting that these targets are not controlled by eIF4A in these NSCLC cells (Figure 5A, heatmap of protein expression; Supplemental Figure 3, Western blots). Notably, the observation that RAS pathway genes (e.g., RTKs, RAS) and downstream effectors (e.g., ERK, phospho-ERK) were not suppressed is consistent with the lack of enhanced suppression of RAS signaling throughout this study and suggests that eIF4A inhibitors do not potentiate the effects of KRAS G12C inhibitors in NSCLC by triggering a deeper suppression of this pathway. Based on these findings, MCL1, BCL-xL, BCL-2, cyclin D1, and CDK4 were selected for further study in additional cell lines.

### eIF4A and RAS/ERK pathway inhibitors fail to suppress the expression of MCL1, BCL-xL, or BCL-2 in resistant cells.

The expression of MCL1, BCL-xL, BCL-2, cyclin D1, and CDK4 was evaluated in response to single and combined agents in multiple sensitive and resistant cell lines. Importantly, the RAS/ERK pathway is known to converge on several of these same targets and can also control their expression by affecting transcription (e.g., cyclin D1) or protein stability (e.g., MCL1) (26, 27). Therefore, in many instances, KRAS G12C inhibitors further enhanced the suppressive effects of eIF4A inhibitors, resulting in a cooperative decrease in many of these target proteins in *KRAS G12C*–mutant cells, with some variation observed between cell lines (Figure 5B). Trametinib and eFT226 exerted similar effects in 2 additional *KRAS*-mutant cell lines (a total of 5) (Figure 5C). Of note, in all sensitive lines, at least 2 and often all 3 prosurvival proteins were potently suppressed by these agents (Figure 5, B and C). However, responses were substantially different in resistant cell lines (Figure 5D). Whereas combined eIF4A/KRAS G12C inhibitors suppressed cyclin D1 and CDK4 in both resistant lines (H1373, H2030), none of the prosurvival proteins (MCL1, BCL-xL, and BCL-2) were substantially suppressed in resistant cells (Figure 5D). These observations raised the intriguing possibility that eIF4A inhibitors might synergize with KRAS G12C inhibitors specifically by suppressing the expression of prosurvival BCL-2 family proteins. Conversely, their maintained expression in insensitive cell lines might contribute to resistance.

### Suppression of multiple prosurvival proteins, but not other oncogenic targets, underlies the therapeutic response to combined eIF4A and KRAS G12C inhibitors.

**Figure 6:**
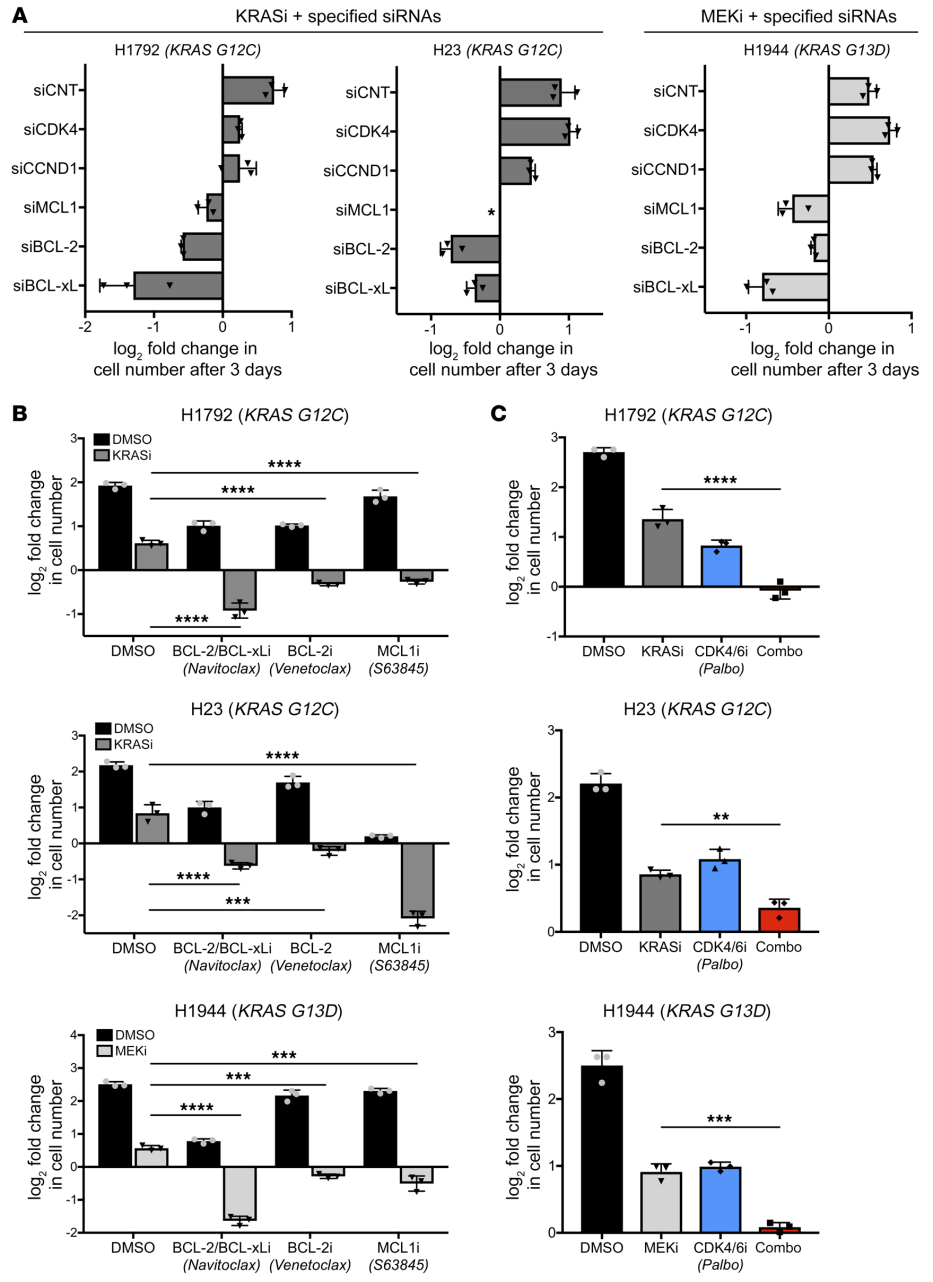
Suppression of prosurvival proteins underlies the therapeutic response to combined eIF4A and RAS pathway inhibitors. (**A**) Bar graphs depicting the effects of either KRASi (left, middle) or MEKi (right) in sensitive cell lines in the presence of the indicated siRNA pools. The fold change in cell number was calculated after 72 hours of treatment (versus day 0) in response to 100 nM MRTX849 (KRASi) or 50 nM trametinib (MEKi). Data are represented as means ± SD. *n* = 3. The siCDK4 studies were performed separately; however, control values were similar (primary data are shown in Supplemental Figure 4). *Complete genetic ablation of MCL1 alone in H23 cells resulted in cell death, preventing further analysis. (**B**) Bar graphs depicting fold changes in cell numbers in cells treated for 72 hours (versus day 0) with 100 nM S63845 (MCL1i) or 1 μM navitoclax (BCL-xL/BCL-2i) or 1 μM venetoclax (BCL-2i) combined with either 100 nM MRTX849 (KRASi) or 50 nM trametinib (MEKi). Data are represented as means ± SD. *n* = 3. ***P < 0.001; ****P < 0.0001, 1-way ANOVA and Tukey’s post hoc test. (**C**) Bar graphs depicting fold change in cell number in cells treated for 72 hours with 500 nM palbociclib (CDK4/6i) combined with either 100 nM MRTX849 (KRASi) or 50 nM trametinib (MEKi). Data are represented as means ± SD. *n* = 3. **P < 0.01; ***P < 0.001; ****P < 0.0001, 1-way ANOVA and Tukey’s post hoc test.

To functionally interrogate individual eIF4A-regulated targets, siRNAs were used to inhibit their expression in the presence and absence of KRAS G12C or MEK inhibitors. Specifically, we sought to determine whether the ablation of any of these eIF4A targets could recapitulate the effects of eFT226 in this context. When combined with KRAS G12C or MEK inhibitors, the acute loss of either CDK4 or cyclin D1 resulted in enhanced cytostasis (H1792 cells) or had no effect (H1944 and H23 cells) (Figure 6A; all data points and Western blots are shown in Supplemental Figure 4). In contrast, the suppression of BCL-2 family genes potently cooperated with MRTX849 or trametinib to kill cells (Figure 6A and Supplemental Figure 4). Specifically, when combined with either KRAS G12C or MEK inhibitors, the ablation of each BCL-2 family member individually exerted a cytotoxic response, although the depth of the response varied between genes and cell lines (Figure 6A and Supplemental Figure 4). These observations are consistent with previous studies demonstrating the heterogeneous dependency on specific survival proteins in NSCLC (28). Notably, these data also directly illustrate potent effects of BCL-2 family protein suppression versus the modest phenotypes conferred by suppression of CDK4 or cyclin D1 when combined with MEK or KRAS G12C inhibitors (Figure 6A).

**Figure 7:**
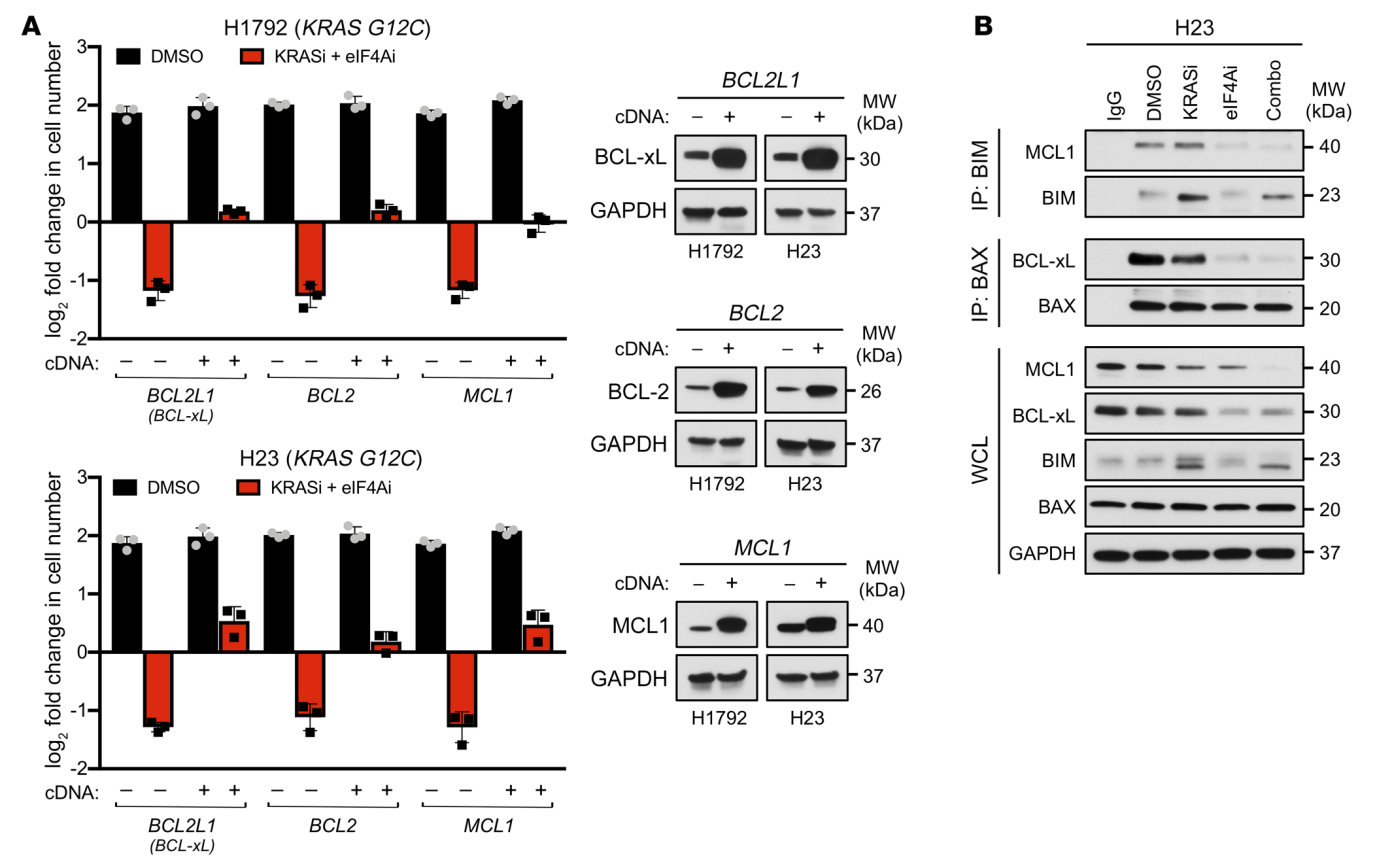
Reconstitution with survival proteins prevents cell death in response to eIF4A and KRAS G12C inhibitors. (**A**) (Left) Bar graphs depicting fold changes in cell numbers of H23 and H1792 cells ectopically expressing *BCL2L1* (BCL-xL), *BCL2*, or *MCL1* cDNAs treated for 72 hours with combined 25 nM eFT226 (eIF4Ai) and 100 nM MRTX849 (KRASi). Data are represented as means ± SD. *n* = 3. (Right) Immunoblots confirming overexpression of BCL-xL, BCL-2, and MCL1 by cDNAs. (**B**) Immunoblots showing interactions of MCL1 and BCL-xL with immunoprecipitated proapoptotic BIM and BAX proteins, respectively, in response to specified drug treatments in sensitive H23 cells.

Similar results were obtained using selective small molecule inhibitors against MCL1 (S63845), BCL-xL/BCL-2 (navitoclax), and BCL-2 (venetoclax). Specifically, these agents triggered cell death and a net loss of cells when combined with either KRAS G12C or MEK inhibitors in H1792 and H23 cells or H1944 cells, respectively (Figure 6B). While genetic ablation exerted more potent effects than chemical inhibition, as expected, different NSCLC cell lines exhibited differential sensitivities to these agents. For example, H1792 cells were most sensitive to BCL-xL/BCL-2 inhibition, H23 cells were most sensitive to MCL1 inhibition, but were also moderately sensitive to BCL-2 inhibition, and H1944 cells were most sensitive to BCL-xL/BCL-2 inhibition, but were also partially sensitive to MCL1 inhibition (Figure 6B). These findings were consistent with the genetic ablation studies shown in Figure 6A. In contrast, the CDK4/6 inhibitor palbociclib only enhanced the cytostatic effects of KRAS G12C or MEK inhibitors and did not promote a loss of cells over time in any cell line (Figure 6C). Therefore, we conclude that, while suppression of CDK4 and/or cyclin D1 may help arrest cells in the presence of KRAS G12C or MEK inhibitors, the suppression of MCL1, BCL-xL, and/or BCL-2 specifically mediates cell killing. In support of this conclusion, ectopic expression of BCL-xL, BCL-2, or MCL1 prevented cell death triggered by KRAS G12C and eIF4A inhibitors; however, cells still largely remained arrested under these conditions (Figure 7A). Together with expression data, these observations suggest that eIF4A and KRAS G12C inhibitors mediate their cytotoxic effects via the suppression of prosurvival BCL-2 family proteins. The observation that eIF4A inhibitor–based drug combinations simultaneously suppress the expression of multiple prosurvival proteins in these tumor cells is particularly important, given that different NSCLCs are thought to be dependent on different or multiple BCL-2 family members (28).

Finally, to confirm that eIF4A inhibitors directly affect apoptotic signaling in NSCLCs, we assessed the dynamics of BIM:MCL1 and BAX:BCL-xL complexes in response to KRAS G12C and eIF4A inhibitors. We utilized H23 cells, which are sensitive to both MCL1 and BCL-xL suppression in the presence of KRAS G12C inhibitors (Figure 6, A and B). As expected, the KRAS G12C inhibitor triggered an accumulation of BIM (Figure 7B, whole-cell lysate [WCL]). Moreover, in this setting, BIM was bound to MCL1, suggesting that MCL1 was restraining its proapoptotic effects (Figure 7B, BIM IP). eIF4A inhibition reduced MCL1 protein levels, but maximal loss of MCL1 required both agents, consistent with observations in Figure 5B (Figure 7B, WCL). More importantly, the addition of eIF4A inhibitors to KRAS G12C inhibitors resulted in the loss of BIM:MCL1 complexes, suggesting that BIM was no longer restrained by MCL1 (Figure 7B, BIM IP).

KRAS G12C inhibitors did not affect the expression of BAX, which was constitutively expressed (Figure 7B, WCL). However, BAX:BCL-xL complexes were readily detected in cells treated with KRAS G12C inhibitors (Figure 7B, BAX IP). Notably, eIF4A inhibition alone was sufficient to suppress BCL-xL expression (Figure 7B, WCL), but more importantly, because of this suppression, BAX:BCL-xL complexes were no longer detected in cells treated with combined eIF4A/KRAS G12C inhibitors (Figure 7B, BAX IP). The dynamics of BIM:MCL1 and BAX:BCL-xL complexes in response to KRAS G12C and eIF4A inhibitors were assessed also in resistant cells. Consistent with the results from Figure 5D, combined eIF4A/KRAS G12C inhibitors did not suppress the levels of the prosurvival proteins MCL1 and BCL-xL (Supplemental Figure 5, WCL). Moreover, no changes in the BIM:MCL1 and BAX:BCL-xL interactions were observed in response to the drug combination (Supplemental Figure 5, BIM IP and BAX IP, respectively). Taken together, these results demonstrate that combined eIF4A and KRAS G12C inhibitors trigger apoptosis by suppressing MCL1 and BCL-xL expression, thereby preventing the formation of BIM:MCL1 and BAX:BCL-xL complexes. The observation that these agents affect the expression of multiple prosurvival proteins in NSCLC also explains the relatively broad efficacy that they have in this tumor type, which is known to exhibit a heterogeneous dependency on BCL-2 family proteins (28). It should be noted that the combined effects of eIF4A and KRAS G12C inhibitors were at least as potent, if not more so, than the effects of BCL-xL/BCL-2 and KRAS G12C inhibitors, whereas eIF4A inhibitors and navitoclax were less effective (Supplemental Figure 6). This was expected because RAS pathway suppression also induces the expression of the proapoptotic protein BIM (Figure 7B). Consistent with this observation, the triple combination of eIF4A, BCL-xL/BCL-2, and KRAS G12C inhibitors exerted the strongest cytotoxic effects (Supplemental Figure 6). However, the point of this study is not to highlight the effects of the triple combination, which would not likely be tolerated, but rather to provide an alternative promising combination (eIF4Ai+KRASi) in NSCLC.

### MYC is amplified and/or overexpressed in tumor cells that are sensitive to eIF4A and RAS pathway inhibitors.

**Figure 8:**
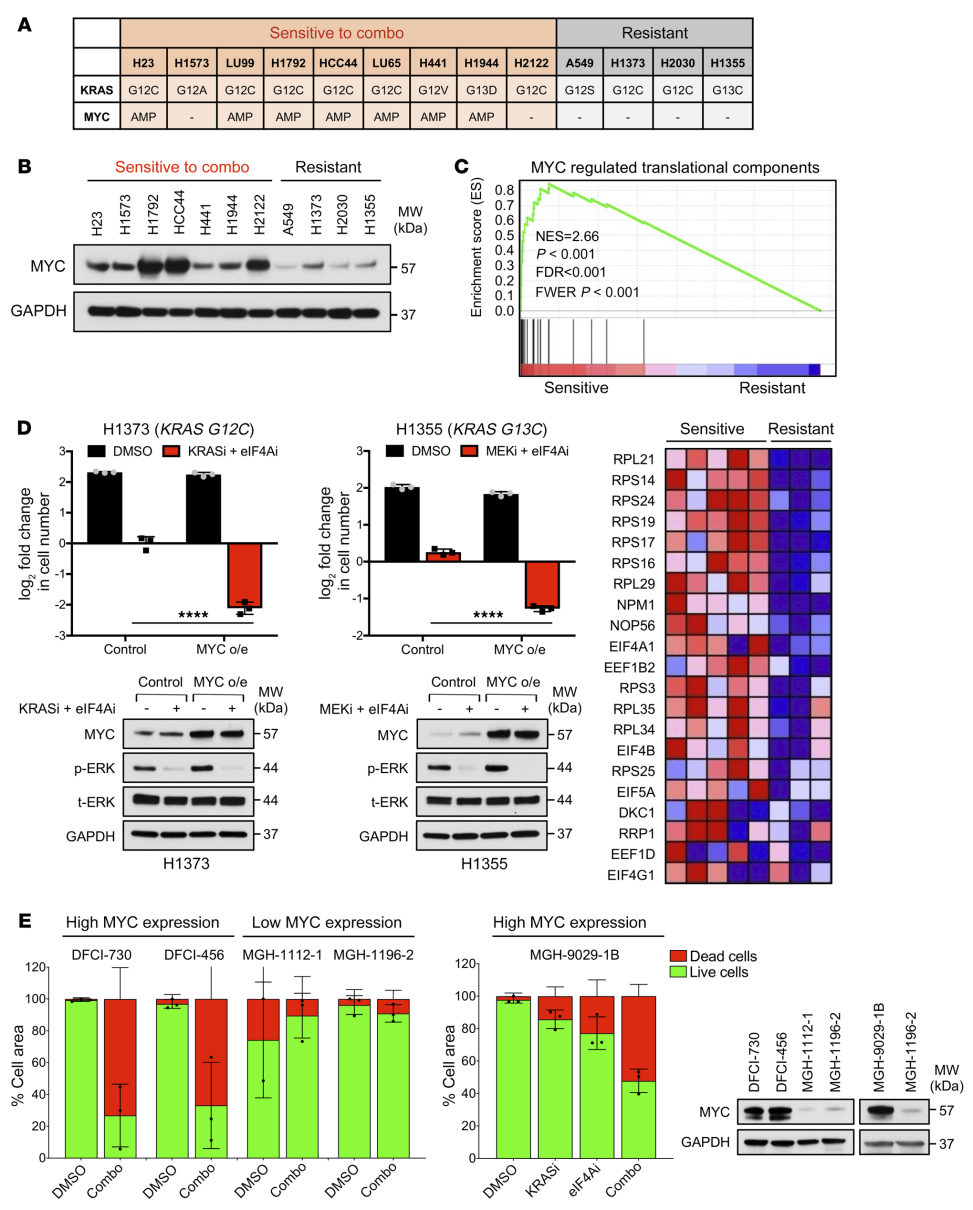
*MYC* amplification or overexpression dictates the sensitivity to combined eIF4A and RAS pathway inhibitors. (**A**) *MYC* copy-number variations (CNV) and *KRAS* mutational data of the 13 NSCLC cell lines retrieved from cBioPortal. (**B**) Immunoblots showing protein expression of MYC in NSCLC cells under baseline conditions. (**C**) GSEA analysis comparing the expression of MYC-regulated ribosomal and translation components in NSCLC cell lines at baseline. Heatmap shows expression of individual genes. (**D**) (Top) Bar graph depicting fold change in cell number of resistant NSCLC cells ectopically expressing control or *MYC* cDNAs treated for 72 hours with vehicle (DMSO) or combined 25 nM eFT226 (eIF4Ai) and either 100 nM MRTX849 (KRASi) or 50 nM trametinib (MEKi). Data are represented as means ± SD. *n* = 3. ****P < 0.001, 1-way ANOVA and Tukey’s post hoc test. (Bottom) Immunoblots showing suppression of phospho-ERK and overexpression of MYC in response to specified treatments and *MYC* ectopic expression. o/e, overexpression. (**E**) (Left) Percentage live/dead of high MYC (DFCI-730, DFCI-456, MGH-9029-1B) and low MYC (MGH-1112-1, MGH-1196-2) PDOTSs treated with DMSO, 25 nM eFT226 (eIF4Ai), 100 nM MRTX849 (KRASi), or combined drugs for 6 days in 3D microfluidic culture. (Right) Immunoblots showing protein levels of MYC in PDX tumor samples under baseline conditions.

As shown in Figure 1A and Figure 2A, 75% of *KRAS*-mutant NSCLC cell lines evaluated were sensitive to combined eIF4A and RAS pathway inhibitors, although a subset were resistant. To identify potential biomarkers of sensitivity or resistance, mutation and copy-number variation data were retrieved from the Cancer Cell Line Encyclopedia (CCLE) data set using cBioPortal (http://www.cbioportal.org). Notably, *MYC* amplifications were present in 7 of 9 sensitive cell lines and in 0 of 4 resistant cell lines (Figure 8A). However, Western blot analysis further revealed a dramatic difference in the expression of MYC protein, which was much more highly expressed in sensitive versus resistant lines, including the 2 sensitive lines that lacked *MYC* amplifications (Figure 8B). Importantly, MYC is known to play a major role in ribosome biogenesis and protein synthesis, in part by upregulating the expression of protein and RNA components of ribosomes as well as transfer RNA (tRNA) (29–32). Moreover, MYC also directly enhances cap-dependent translation by increasing the transcription of multiple eIF4F components and by promoting mRNA capping (29–32). Therefore, we hypothesized that NSCLCs that overexpress MYC may have evolved to harbor a dependency on the eIF4F translational machinery for the expression of these prosurvival proteins. It should be noted that *MYC* copy-number gains are observed in 64% of *KRAS*-mutant NSCLCs (56% copy gain, 8% amplification) (Supplemental Figure 7).

To determine whether we could distinguish sensitive versus resistant cell lines based on their broad expression of MYC-regulated components of the translational machinery, gene set enrichment analysis (GSEA) was used to compare the transcriptional profiles of sensitive versus resistant NSCLC cells. Importantly, this analysis revealed that MYC-regulated ribosomal and cap-dependent translational targets were differentially upregulated in sensitive lines as compared with resistant cells (Figure 8C), consistent with the notion that MYC rewires cells to become more dependent on protein translation (29). In this respect, it should be noted that MYC does not upregulate a single gene involved in protein translation, but rather multiple components.

### MYC overexpression sensitizes cells to eIF4A and RAS pathway inhibitors.

To experimentally determine whether MYC overexpression actively confers sensitivity to eIF4A/RAS pathway inhibitors, *MYC* was ectopically expressed in all 4 resistant lines. Cells were cultured for 2 or more weeks to permit the potential development of a MYC-dependent translational dependency. Strikingly, while control cells remained resistant to the drug combinations, ectopic expression of *MYC* caused all 4 resistant lines to become sensitive to eIF4A/KRAS G12C or eIF4A/MEK inhibitor combinations (Figure 8D and Supplemental Figure 8). Immunoblots confirmed ectopic expression of MYC as well as the suppression of ERK phosphorylation by KRAS G12C or MEK inhibitors (Figure 8D and Supplemental Figure 8).

In addition, to investigate whether high MYC levels might be useful for prospectively predicting sensitivity to combined KRAS G12C/eIF4A inhibitors, human *KRAS G12C*–mutant NSCLC explants expressing either high levels of MYC (DFCI-730, DFCI-456, MGH-9029-1B) or low levels of MYC (MGH-1112-1, MGH-1196-2) were used to generate patient-derived organotypic tumor spheroids (PDOTSs) (33). The sensitivity to combined eIF4A and KRAS G12C inhibitors ex vivo in 3D microfluidic cultures was evaluated, using previously established methods (33). Importantly, we found that tumors with high levels of MYC were sensitive to these agents, whereas those with low levels of MYC were resistant (Figure 8E). Notably, cell death in response to all 4 treatment arms in organoids recapitulates what we observed in cell lines in vitro and in xenografts and PDX tumors in vivo (Figure 8E). Together, these studies suggest that MYC overexpression plays a critical functional role in sensitizing *KRAS*-mutant NSCLCs to combined eIF4A and RAS/ERK pathway inhibitors and may also represent a predictive biomarker for these two promising therapeutic combinations in NSCLCs.

### MYC overexpression creates a dependency on eIF4A for the expression of prosurvival proteins.

**Figure 9:**
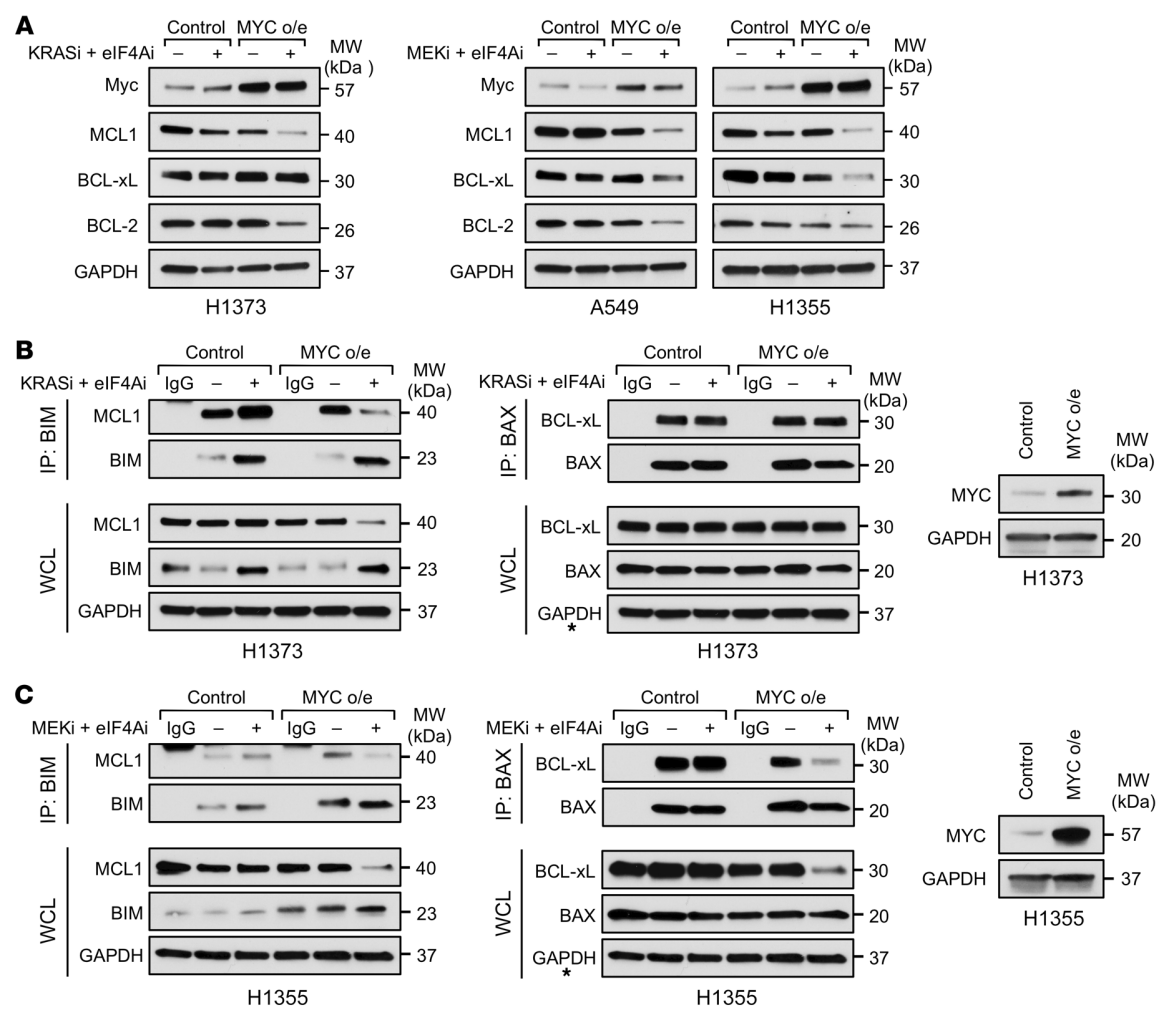
MYC overexpression creates a dependency on eIF4A for the expression of prosurvival proteins. (**A**) Immunoblots showing MCL1, BCL-xL, and BCL-2 protein levels in response to 24 hours of specified treatments in control and MYC-overexpressing resistant lines. (**B** and **C**) Immunoblots showing interactions of MCL1 and BCL-xL with immunoprecipitated proapoptotic BIM and BAX proteins, respectively, in response to specified drug treatments in control and MYC-overexpressing resistant lines. The GAPDH immunoblots denoted by asterisks, which serve as a loading control, have been duplicated from the left side of that panel, because immunoblots were all generated from the same gel and membrane. (Right) Immunoblots confirm ectopic expression of MYC by cDNAs.

As shown in Figure 5D, the expression of the BCL-2 family proteins was not affected by combined eIF4A and KRAS G12C inhibitors in resistant cell lines. Therefore, we sought to determine whether MYC overexpression could rewire translation to create a new dependency on eIF4A/F for the expression of these prosurvival proteins. Importantly, we found that, while the expression of MCL1, BCL-2, or BCL-xL in resistant (control) cell lines was not affected by eIF4A/KRAS G12C or eIF4A/MEK inhibitor combinations, these agents potently suppressed 2 or more of these prosurvival proteins in cell lines that had adapted to MYC overexpression (Figure 9A).

Consistent with these findings, MYC overexpression in H1373 cells decreased the amount of MCL-1 bound to BIM in response to these agents (Figure 9B). MYC overexpression also suppressed BIM:MCL-1 complexes in H1355 cells, but had a more dominant effect on suppressing BCL-xL and consequently BCL-xL:BAX complexes (Figure 9C). These observations are consistent with findings shown in Figure 9A and illustrate how these agents are able to kill NSCLCs even with heterogeneous BCL-2 protein expression/dependency. They further demonstrate that MYC overexpression creates a new dependency on eIF4A/F for the expression of these important prosurvival proteins.

## Discussion

KRAS G12C inhibitors have recently been approved for the treatment of NSCLCs (34). Importantly, their development represents a major therapeutic advance that will likely extend to other *KRAS G12C–*mutant tumor types. Nevertheless, modified strategies that confer more effective and durable responses are urgently needed. In this study, we show that eIF4A inhibitors dramatically sensitize NSCLCs to KRAS G12C inhibitors. Together, these agents synergize, substantially enhance apoptosis, and trigger potent tumor regression in vivo, even in models that are relatively insensitive to KRAS G12C inhibition alone. We further show that eIF4A inhibitors cooperate with MEK inhibitors in other *KRAS*-mutant NSCLCs and similarly promote the regression of tumors that do not respond to MEK inhibitors as single agents. Collectively, these studies reveal two promising therapeutic strategies for *KRAS-*mutant NSCLCs.

The RNA helicase eIF4A unwinds the complex 5′ UTR structures of cap-dependent transcripts and is a critical component of the eIF4F translational complex (10, 17). Many direct eIF4A/eIF4F mRNA targets have been identified and often encode proteins that drive proliferation, metastasis, and survival (11, 14, 15). Nevertheless, these targets appear to be very context and/or tissue-type specific, in that eIF4A/F-mediated translation limits the expression of specific transcripts in some settings but not others. In this study, we screened a broad panel of direct eIF4A/F targets to identify those that specifically enhance the effects of KRAS G12C inhibitors in NSCLCs. We found that eIF4A inhibitors suppress the expression of a defined subset of eIF4A/F targets in *KRAS-*mutant NSCLCs; however, only the suppression of prosurvival BCL-2 family proteins was necessary and sufficient to promote cell death when combined with KRAS G12C or MEK inhibitors. In contrast, the suppression of other targets, such as cyclin D1 and CDK4, enhanced the cytostatic effects of KRAS G12C or MEK inhibitors in some cell lines, but did not promote cell killing. Together, these studies have deconstructed the mechanism by which these agents function in NSCLCs and identified the key component(s) of the therapeutic response.

Several findings in this study warrant further emphasis. First, in many instances, we found that eIF4A and KRAS G12C or MEK inhibitors cooperatively suppressed BCL-2 family proteins. This observation is consistent with reports that the RAS/MEK/ERK pathway also regulates many of these same genes via transcriptional and posttranslational mechanisms (27, 35–37). Notably, this convergence may be responsible for the observed synergy between these agents. It may also reduce the need for deeper RAS pathway suppression, which could ultimately prove to be too toxic.

Second, in all sensitive NSCLC lines, combined KRAS G12C and eIF4A inhibitors suppressed the expression of multiple prosurvival BCL-2 family proteins. This finding is particularly important for NSCLCs, as this tumor type has been shown to exhibit a heterogeneous dependency on MCL1, BCL-xL, and BCL-2 (28). Therefore, eIF4A inhibitor–based combinations may be more broadly effective in NSCLCs because they should kill tumors that are dependent on MCL1, BCL-xL, BCL-2, or multiple BCL-2 family proteins. In addition, eIF4A inhibitors may also have a greater therapeutic index than BH3 mimetics because they target the enhanced translation of BCL-2 family proteins in tumor cells rather than the biological activity of these proteins in all cells.

Finally, we report that MYC overexpression dictates the sensitivity to these combinations. Notably, MYC is a well-established regulator of ribosome biogenesis and protein translation, including cap-dependent translation, and can rewire translational control in tumor cells when it is overexpressed (29–31). Here, we show that *MYC* is amplified and/or overexpressed in cells that are sensitive to RAS pathway/eIF4A inhibitors and that BCL-2 family proteins are not inhibited by eIF4A inhibitors in resistant NSCLCs. More importantly, however, MYC overexpression causes a shift in dependency on eIF4A/eIF4F. Specifically, MYC overexpression sensitizes resistant cells to these combinations by causing the expression of these prosurvival proteins to be dependent on eIF4A. These conclusions are supported by functional data generated from 13 cell lines, in vivo tumor-regression studies, and the analysis of primary human tumor tissues. As previously reported and shown herein, MYC induces the expression of a plethora of genes that regulate protein translation. Therefore, we believe that the shift in translational dependency is due to the combined effects on many of these components and not one specific target.

Thus, collectively, these studies identify 2 promising therapeutic strategies for *KRAS*-mutant NSCLCs, identify the critical mediators of the therapeutic response, and uncover a functional biomarker that predicts treatment sensitivity. Accordingly, while approaches aimed at promoting a deeper vertical inhibition of the RAS pathway represent one therapeutic option, these findings provide an alternative strategy that does not rely on deeper pathway suppression, which may offer a unique therapeutic window.

## Methods

### Cell lines and drug treatments.

All human *KRAS*-mutant NSCLC cell lines were purchased from ATCC, except for LU99 and LU65, obtained from JCRB Cell Bank, and H2122, obtained in house. Cells were maintained in RPMI 1640 medium supplemented with 10% FBS and 1% streptomycin/penicillin at 37°C under 5% CO_2_. Human cell-line authentication was performed through short tandem repeat (STR) marker genotyping. All cell lines tested negative for *Mycoplasma* contamination using the MycoAlert Detection Kit (Lonza, catalog LT07-318). For drug treatments, eIF4A inhibitor eFT226 and KRAS G12C inhibitor MRTX849 were purchased from MedChemExpress; MEK inhibitor trametinib was purchased from LC Laboratories; BCL-xL/BCL-2 inhibitor navitoclax, BCL-2 inhibitor venetoclax, and CDK4/6 inhibitor palbociclib were purchased from Selleckchem; and MCL1 inhibitor S63845 was purchased from Chemietek.

### Short-term cell-proliferation assays.

For short-term cell-proliferation assays, 70,000–100,000 cells/well were plated in triplicate in 6-well standard tissue-culture plates. After approximately 24 hours, cells were counted for day 0 time points using a hemocytometer. Immediately following day 0 counts, drug treatment was started for 3 days. Final cell counts were taken 72 hours after day 0 to determine changes in cell number compared with zero time points. For 3D tissue-culture conditions, 150,000 cells/well were seeded in nonadherent ULA 6-well plates, which favors the formation of spheres. Cells were then counted at day 0 and after 72 hours of treatment. At end point (72 hours), images were taken using the 2X Bright Field channel. Proliferation experiments that included siRNA knockdown were performed on cells approximately 24 hours after the initial transfection.

### Long-term cell-growth assays.

For long-term cell-growth assays, cells were seeded at 20,000-30,000 cells/well in 12-well plates and treated up to 3 weeks with vehicle, single agents, or drug combinations in 2% FBS media. At days 7, 14, and 21, cells were fixed in 10% formalin for 15 minutes and stained with 0.02% crystal violet for 1 hour.

### Synergy score analysis.

To measure synergistic interactions of eIF4A inhibitor combined with either KRAS G12C or MEK inhibitors, cells were seeded in 96-well white flat-bottom plates at 3,500 cells/well (H23, H1792, H1573, and H1944) and 1,000 cells/well (HCC44). Three technical replicates were done for each condition. After 24 hours, drugs were added at the following concentrations: eIF4A inhibitor (0, 5, 10, 25, 50 nM), KRAS G12C inhibitor (0, 10, 50, 100, 1000 nM), and MEK inhibitor (0, 10, 25, 50, 100 nM). After 72 hours of treatment, cell viability was quantified using CellTiter-Glo (Promega, catalog G9291) according to the manufacturer’s protocol. From the resulting raw CellTiter-Glo assay readout, the triplicate values for each condition were averaged and normalized to the DMSO, and the final inhibitory response was calculated. SynergyFinder was then used to calculate the synergy score using the HSA model where a value greater than 10 indicates a synergistic interaction.

### Incucyte live-cell imaging.

To create stable cell lines with red-stained nuclei, cells were infected with Incucyte Nuclight Red (Sartorius, catalog 4625) and selected in puromycin. Stably transfected cells were then seeded in 96-well black clear-bottom plates at 3,500 cells/well and allowed to settle overnight. Three technical replicates were done for each condition. After 24 hours, the growth medium was replaced with medium containing green caspase-3/7 apoptosis reagent (Biotium, catalog 10402) at 1:1,000 dilution and vehicle or appropriate drug treatments. Plates were placed in the Incucyte machine, and images were acquired every 2 hours over the course of 5 days to assess real-time quantification of cell death. Using the Incucyte integrated analysis software, red and green fluorescent objects were counted. Specifically, cell death was determined by the presence of yellow cells containing green signal (caspase-3/7 reagent) overlapping with red signal (nuclei). Four images were taken per well, and triplicate wells were counted and averaged per condition.

### Transfections and infections.

For transfections, cells were incubated for 6 hours with 10 μM siRNA constructs using a 1:400 dilution of Lipofectamine RNAiMAX Transfection Reagent (Invitrogen, catalog 13778075) in antibiotic-free media. Nontargeting, eIF4A1, MCL1, BCL-xL, BCL-2, cyclin D1, and CDK4 siRNA ON-TARGETplus smart pools were purchased from Dharmacon (catalog D-001810-10-50, L-020178-00-0005, L-004501-00-0005, L-003458-00-0005, L-003282-02-0005, L-003210-00-0005, and L-003238-00-0005, respectively). For infection experiments, cDNA constructs were prepared, and virus was harvested as previously described (38). Virus was then incubated on target cells for 8 to 20 hours at a 1:5 dilution with 8 mg/mL polybrene. Infected cells were allowed to recover for 24 hours and then selected with 0.5–2.0 μg/mL puromycin. Selection continued until uninfected control cells were dead, for approximately 3 to 4 days depending on the cell line. *BCL2L1-pLX307* and *pCDH-puro-BCL-2* lentiviral expression vectors were purchased from Addgene (catalog 98323, and 46971, respectively). *pCDH-PURO-cMYC* lentiviral expression vector was obtained from Kris C. Wood (Duke University, Durham, North Carolina, USA). *MCL1* cDNA was purchased from the DNASU Plasmid Repository (catalog HsCD00042645) and cloned into a lentiviral *N-HA-FLAG-pHAGE* vector provided by Wade Harper (Harvard Medical School, Boston, Massachusetts, USA).

### Western blot and immunoprecipitation assays.

Cells were lysed in 1% SDS lysis buffer, and protein concentrations were determined and normalized using the Pierce BCA Protein Assay (Thermo Fisher Scientific, catalog 23224, 23228). The following primary antibodies were used for detection: ERK1/2 (catalog 9102), phospho-ERK1/2 (catalog 4370), MCL1 (catalog 39224), BCL-xL (catalog 2764), BCL-2 (catalog 4223), BIM (catalog 2819), BAX (catalog 2772), AKT (catalog 9272), phospho-AKT (catalog 4060), PI3K (catalog 4255), MET (catalog 3127), HER2 (catalog 2242), YAP (catalog 14074), CDK4 (catalog 12790), eIF4A1 (catalog 2490), GAPDH (catalog 2118) (purchased from Cell Signaling Technologies). c-MYC (catalog ab32072) was purchased from Abcam. Cyclin B1 (catalog sc-245), cyclin E (catalog sc-377100), and KRAS (catalog sc-30) were purchased from Santa Cruz Biotechnology Inc. Cyclin D1 (catalog RB-010) was purchased from Thermo Fisher Scientific. For immunoprecipitation studies, cells were lysed in NP40 lysis buffer with the addition of PhosSTOP Phosphatase Inhibitor Cocktail (Roche, catalog 04906837001) and cOmplete Mini Protease Inhibitor Cocktail (Roche, catalog 11836153001). Total cell lysate (350 μg) was incubated with BIM antibody at a 1:200 dilution (Cell Signaling Technologies, catalog 2819), BAX antibody at a 1:50 dilution (Cell Signaling Technologies, catalog 2772), or normal rabbit IgG (Millipore, catalog 12-370) overnight at 4°C under rotation. The following day, 50 μL of protein A/G magnetic beads (Thermo Fisher Scientific, catalog 88803) were washed and incubated with lysates for 1 hour at 4°C under rotation. Beads were then collected and washed with lysis buffer; proteins were elute at room temperature for 15 minutes in 2× sample buffer. For each condition, the immunoprecipitate and WCL were analyzed by Western blot.

### Xenograft models and in vivo drug treatments.

For cancer cell xenograft models, athymic nu/nu mice 6 to 8 weeks of age were purchased from Charles River Laboratory, and 3.5 × 10^6^ cells (H23 or H1573) and 5 × 10^6^ cells (H441 and H2030) were resuspended in 50:50 Matrigel/media and injected into the flanks of each mouse. For the PDX model, in collaboration with the Belfer Center, we obtained the DFCI-730 tumor sample derived from a surgical biopsy of a patient with *KRAS G12C*–mutant lung adenocarcinoma. DFCI-730 was initially implanted into the subrenal capsule of mice for expansion. After initial implantation, DFCI-730 tumors were expanded and passaged repeatedly in mice as subcutaneous tumors. Tumors used in efficacy studies were implanted subcutaneously after dipping in Matrigel in 8- to 10-week-old female NSG (NOD.Cg-Prkdc^scid^ Il2rg^tm1WjI^/SzJ) mice purchased from Jackson Laboratory (catalog 005557). Tumor length and width were measured with vernier calipers, and tumor volumes were calculated using the standard formula: L × W^2^ × 0.52. Once tumors formed, mice were randomly enrolled into 1 of the 4 treatment arms: vehicle, drug 1 (eIF4A inhibitor), drug 2 (KRAS G12C or MEK inhibitors), and drug combination (eIF4A/KRAS G12C or eIF4A/MEK inhibitors). Specifically, eIF4A inhibitor eFT226 was administered at 0.5 mg/kg via intraperitoneal injection (i.p.) Q4D and prepared in 5% dextrose in water. KRAS G12C inhibitor MRTX849 was administered at 100 mg/kg via oral gavage (OG) daily and prepared in 10% Captisol in 50 mM citrate buffer, pH 5.0. MEK inhibitor trametinib was administered at 0.6 mg/kg via OG daily and prepared in 0.5% hydroxypropyl methylcellulose, 0.2% Tween-80, pH 8.0. To track changes in tumor volume, tumor size was blindly measured at day 0 and subsequently 2 to 3 times per week for a total of 28 days of treatment. During the entire treatment window, animals were evaluated for signs of toxicity by monitoring body weight and body condition daily.

### PDOTSs and live/dead analysis.

In collaboration with the Belfer Center and the Massachusetts General Hospital, we obtained 5 tumor samples from patients with *KRAS G12C*–mutant lung adenocarcinoma. PDOTSs were generated as previously described (33). Briefly, fresh human *KRAS*-mutant NSCLC tumor specimens were minced in prewarmed (37°C) full media (RPMI+10% FBS) using a human tumor dissociate kit (Miltenyi Biotec, catalog 130-095-929). Dissociated material was strained over 100 μm and 40 μm filters to generate S1 (>100 μm), S2 (40-100 μm), and S3 (<40 μm) spheroid fractions. S2 fractions were pelleted and resuspended in type I rat tail collagen (Corning, catalog 354236) at a concentration of 2.5 mg/mL. The spheroid-collagen mixture was then injected into the 3D microfluidic culture device (AIM Biotech, catalog DAX-1) and incubated for 30 minutes at 37°C. Finally, collagen hydrogels containing PDOTS were hydrated with media with indicated treatments and cultured for 6 days at 37°C in humidity chambers. For live/dead analysis, microfluidic devices were loaded with Hoechst 33342 (catalog H3570, Invitrogen) and DRAQ7 (catalog 424001, BioLegend) staining and incubated at room temperature in the dark for 20 and 15 minutes, respectively. Image capture and analysis were performed using the NIS-Elements AR software package, version 5.02.01. Live/dead cell quantitation was performed by measuring the total cell area of each dye. The spheroid’s viability was determined by calculating the ratio of dead cells as equal to 100% × DRAQ7/Hoechst and the ratio of live cells as equal to 100% – ratio of dead cells.

### MYC copy-number analysis.

For cell lines, *MYC* copy-number data were accessed via cBioPortal from the CCLE entry. *MYC* allelic copy number was characterized as deletion, diploid, or amplification (39). For human tumor samples, data for lung adenocarcinoma were retrieved from The Cancer Genome Atlas Project Firehose Legacy data set and accessed via Firebrowse (40). 75 *KRAS*-mutant tumor samples were identified using oncotated mutation calls (41). Copy-number analysis data were accessed, and *MYC* copy number was classified based on the GISTIC2 method as deletion, diploid, copy-number gain, or amplification (42).

### GSEA analysis of human lung cancer cell lines.

Publicly available CCLE data were downloaded through the DepMap portal (Public 22Q2 CCLE_RNAseq_reads.csv; https://depmap.org/portal/download/all/?releasename=DepMap+Public+22Q2&filename=CCLE_RNAseq_reads.csv), and read counts were normalized using the DESeq2 module in GenePattern (genepattern.org). To analyze the differential expression of MYC-regulated genes involved in ribosome biogenesis and translation in sensitive versus resistant human lung cancer cell lines, we generated a MYC-regulated translational components signature (*RPL21, RPL29, RPL34, RPL35, RPS14, RPS16, RPS17, RPS19, RPS24, RPS25, RPS3, EIF4G1, EIF4A1, EIF4B, EIF5A, EEF1B2, EEF1D, NPM1, DKC1, NOP56, RRP1*) based on previously published data (29). GSEA was performed using GSEA 4.0.1 software downloaded from gsea-msigbd.org. The heatmap shown was generated in this analysis.

### Statistics.

For quantitative measurements of cell proliferation, graphs represent means of indicated number of replicates ± SD. For quantitative measurements of tumor growth, graphs depict means of indicated numbers of tumors ± SEM. Where indicated, data are presented as log_2_ fold change (left axis) and percentage change (right axis) over initial measurements. Waterfall plots are used to depict changes in individual tumor volumes within the 4 treatment arms. A bar over the zero line indicates tumor growth, and a bar under the zero line indicates tumor shrinkage. All in vitro studies were performed in multiple cell lines 3 or more times with 3 technical replicates for each condition. One-way ANOVA followed by Tukey’s post hoc test for multiple comparisons was used to compare data sets, and *P* values are indicated. A *P* value equal to or less than 0.05 was considered significant. ImageJ software (NIH) was used to quantify the protein levels by densitometry. Data were graphed and analyzed using GraphPad Prism 7.0, except for the synergy data, which were analyzed and visualized using SynergyFinder. Synergy score was calculated using the HSA model to characterize the strength of synergistic interaction between the 2 drugs.

### Study approval.

All xenograft animal procedures were approved by the Center for Animal and Comparative Medicine at Brigham and Women’s Hospital in accordance with the NIH *Guide for the Care and Use of Laboratory Animals* (National Academies Press, 2011) (IACUC) and the Animal Welfare Act (protocol 2016N000467). PDX studies were conducted at Dana-Farber Cancer Institute in accordance with IACUC guidelines in a vivarium accredited by the Association for Assessment and Accreditation of Laboratory Animal Care.

### Data availability.

All data in this study are presented in the article and Supplemental Information. All materials are available upon request and through a material transfer agreement. Inquiries should be directed to the corresponding author. Values for all data points in graphs are reported in the Supporting Data Values file.

## Author contributions

FN, NP, AES, and ZHL, and KN performed the experiments. FN, NP, AES, ZHL, EVI, AS, and KC analyzed the data. EVI, CL, PCG, ANH, DAB, CPP, and PAJ provided expertise and materials. FN and KC designed the experiments and wrote the manuscript.

## Supplementary Material

Supplemental data

Supporting data values

## Version 1 06/29/2023

In-Press Preview

## Version 2 08/15/2023

Electronic publication
