# Supplementary material for: Cotargeting a MYC/eIF4A-survival axis improves the efficacy of KRAS inhibitors in lung cancer
Source: J Clin Invest. 2023 Aug 15;133(16):e167651. doi: 10.1172/JCI167651 (PMC10425214; doi:10.1172/JCI167651)
Supplement: Supplemental data [file jci-133-167651-s049.pdf]

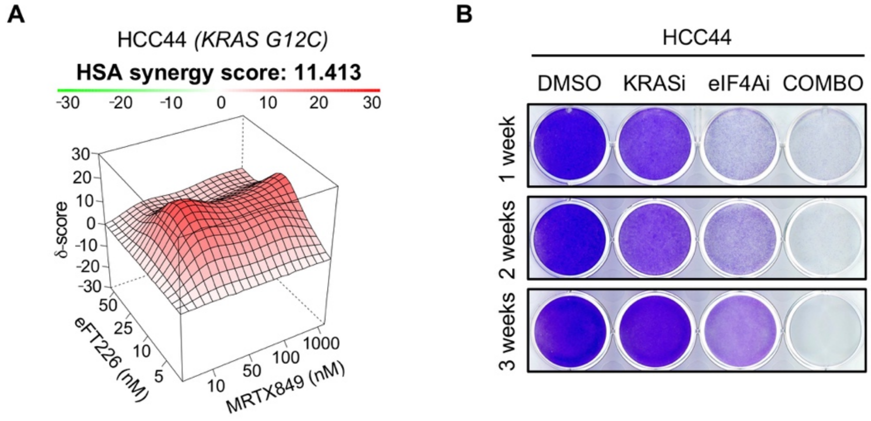
A
HCC44 (KRAS G12C)
HSA synergy score: 11.413
-30 -20 -10 0 10 20 30
δ-score
30
20
10
0
-10
-20
-30
eFT226 (nM)
50
25
10
5
MRTX849 (nM)
10
50
100
1000
B
HCC44
DMSO
KRASi
eIF4Ai
COMBO
1 week
2 weeks
3 weeks

Supplemental Figure 1. eIF4A and KRAS G12C inhibitors synergize and cause durable cytotoxic responses in NSCLC cells. (**A**) Synergy plot depicting the effects of eFT226 and MRTX849 using the HSA model in HCC44 cells. (**B**) Long-term cell proliferation assay of HCC44 cells treated with vehicle (DMSO), 25 nM eFT226 (eIF4Ai), 100 nM MRTX849 (KRASi), or drug combinations (COMBO) up to 3 weeks.

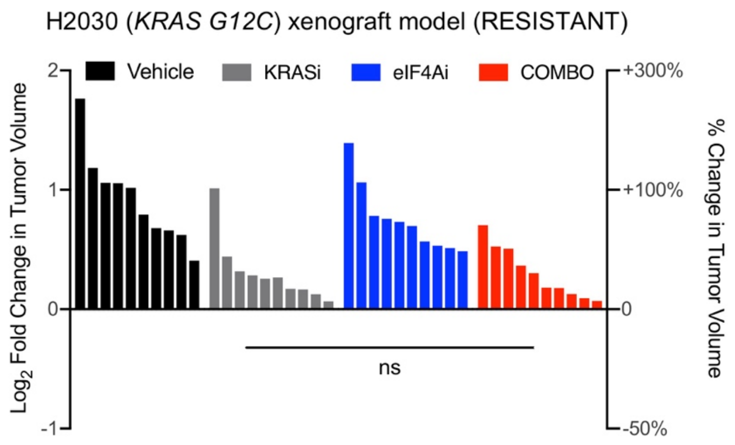

H2030 (KRAS G12C) xenograft model (RESISTANT)
Vehicle
KRASi
eIF4Ai
COMBO
2
1
0
-1
+300%
+100%
0
-50%
Log2 Fold Change in Tumor Volume
% Change in Tumor Volume
ns

Supplemental Figure 2. Combined eIF4A and KRAS G12C inhibitors do not cause tumor regression in resistant xenograft models. Waterfall plot depicting fold-change of each tumor in resistant H2030-derived xenograft models treated for 14 days with vehicle, 100 mg/kg QD MRTX849 (KRASi), 0.5 mg/kg Q4D eFT226 (eIF4Ai), or the two agents together (COMBO) (versus day 0) (ns, not significant; one-way ANOVA and Tukey's post hoc test).

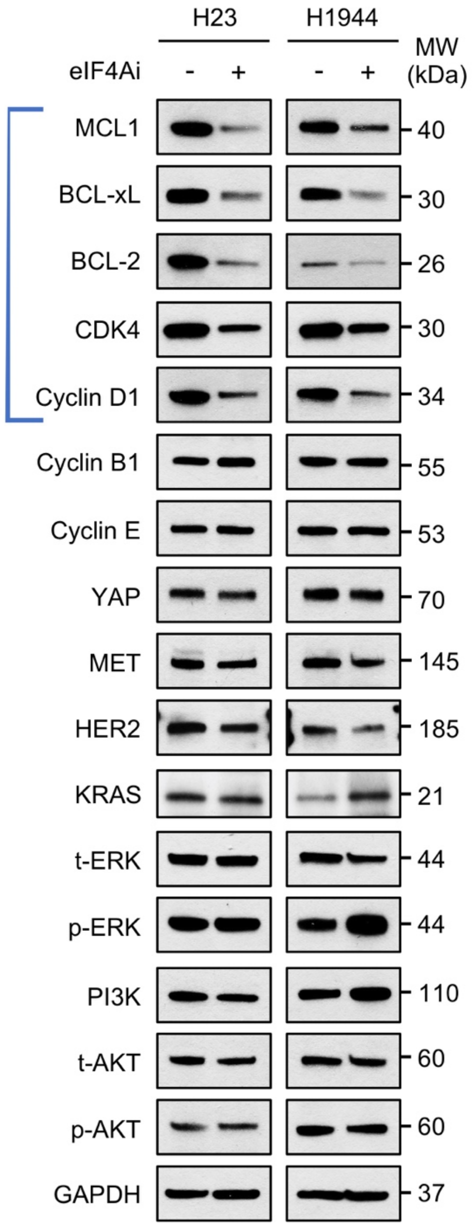

H23
H1944
MW (kDa)
eIF4Ai
-
+
-
+
MCL1
40
BCL-xL
30
BCL-2
26
CDK4
30
Cyclin D1
34
Cyclin B1
55
Cyclin E
53
YAP
70
MET
145
HER2
185
KRAS
21
t-ERK
44
p-ERK
44
PI3K
110
t-AKT
60
p-AKT
60
GAPDH
37

Supplemental Figure 3. eIF4A inhibitors suppress the expression of pro-survival BCL-2 family proteins, Cyclin D1, and CDK4 in sensitive NSCLCs. Immunoblots showing the protein expression of established eIF4A-regulated candidates in H23 and H1944 sensitive lines after 24 hours of treatment with vehicle (DMSO) or eFT226 (eIF4Ai). Bracket indicates the targets to be further investigated.

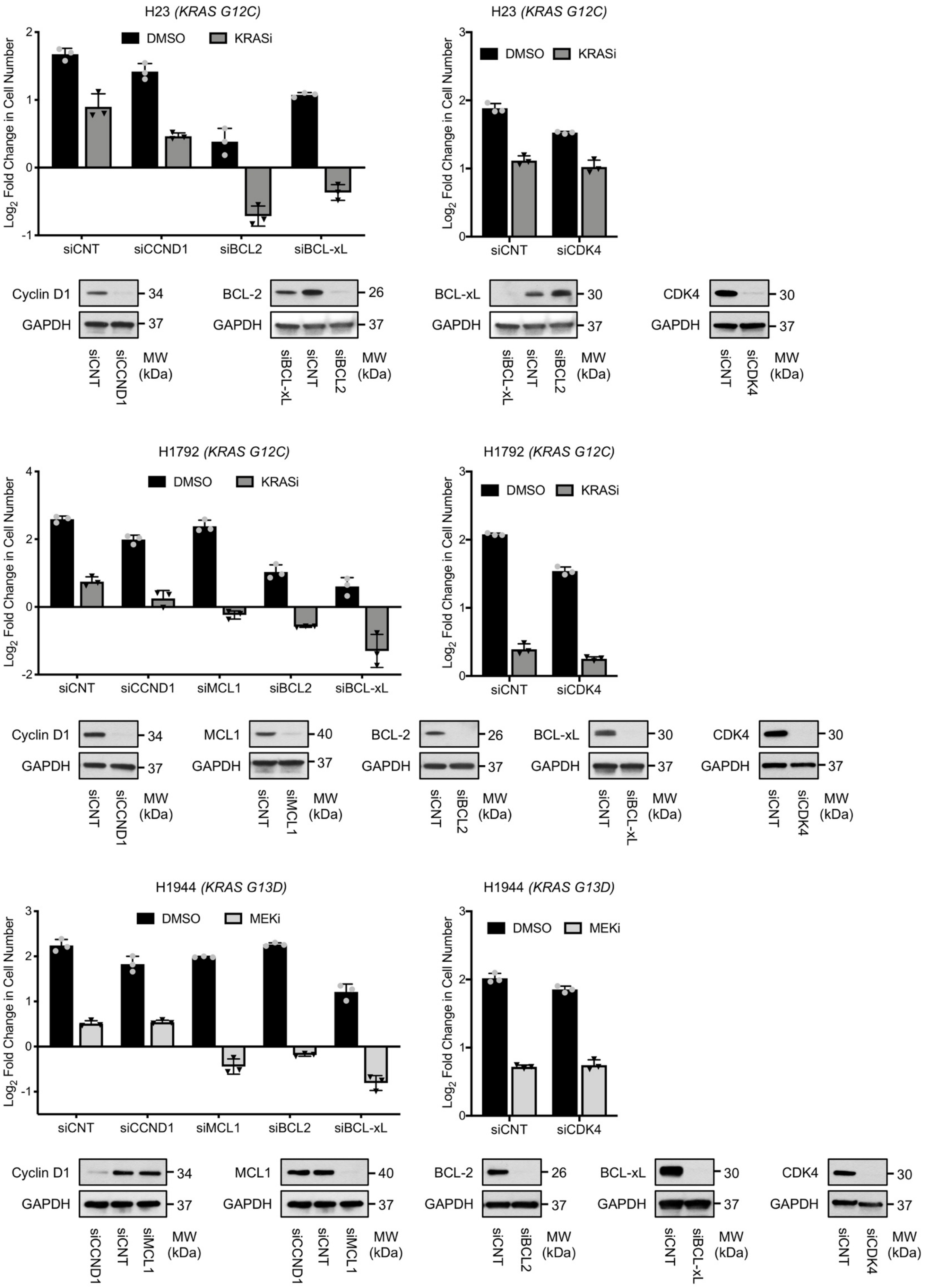

H23 (KRAS G12C)
DMSO
KRASi
Log2 Fold Change in Cell Number
siCNT
siCCND1
siBCL2
siBCL-xL
siCDK4
Cyclin D1
BCL-2
BCL-xL
CDK4
GAPDH
MW (kDa)
H1792 (KRAS G12C)
siMCL1
MCL1
H1944 (KRAS G13D)
MEKi
34
26
30
37
40

Supplemental Figure 4. Suppression of the pro-survival proteins is required to trigger cell death when combined with KRAS G12C or MEK inhibitors. Bar graph depicting fold-change in cell number of sensitive H23, H1792, and H1944 cells where specified genes were individually knocked down via siRNAs and cells were treated for 72 hours with either 100 nM MRTX849 (KRASi) or 50 nM trametinib (MEKi) (mean ± SD, $n = 3$).

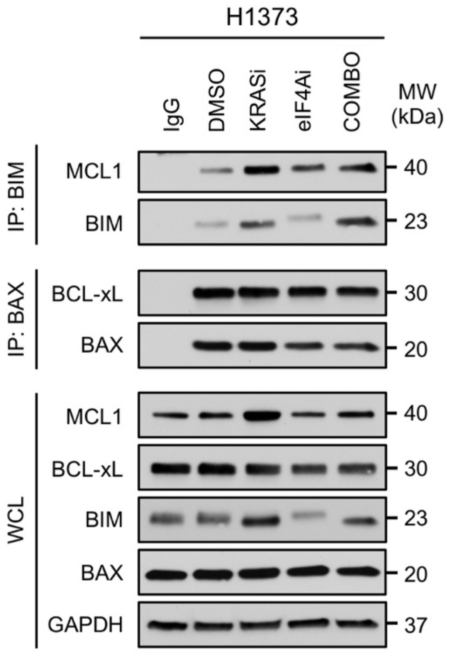

H1373
IgG
DMSO
KRASi
eIF4Ai
COMBO
MW (kDa)
IP: BIM
MCL1
40
BIM
23
IP: BAX
BCL-xL
30
BAX
20
WCL
MCL1
40
BCL-xL
30
BIM
23
BAX
20
GAPDH
37

Supplemental Figure 5. Inhibition of eIF4A does not affect apoptotic signaling in resistant cells. Immunoblots showing interactions of MCL1 and BCL-xL with immunoprecipitated pro-apoptotic BIM and BAX proteins, respectively, in response to specified drug treatments in resistant H1373 cells (WCL, whole cell lysate; IP, immunoprecipitation).

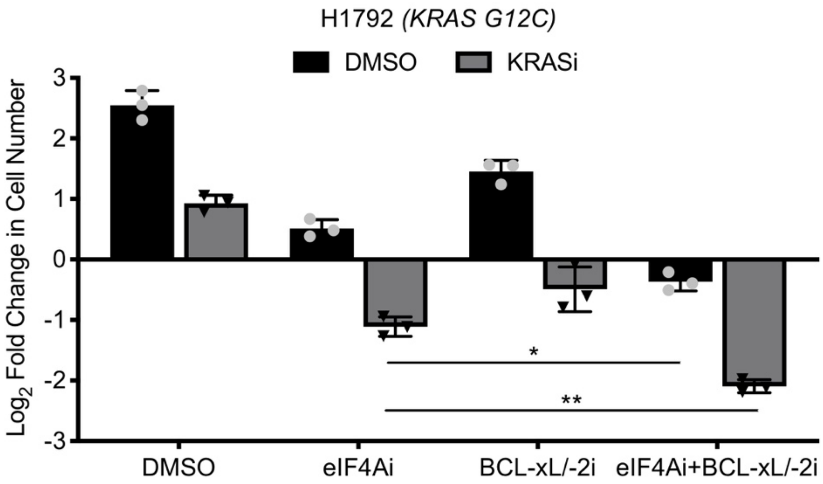
H1792 (KRAS G12C)
DMSO
KRASi
Log2 Fold Change in Cell Number
3
2
1
0
-1
-2
-3
*
**
DMSO
eIF4Ai
BCL-xL/-2i
eIF4Ai+BCL-xL/-2i

Supplemental Figure 6. Relative effects of different eIF4A inhibitor-based combinations. Bar graph depicting fold-change in cell number of H1792 cells treated for 72 hours (versus day 0) with 25 nM eFT226 (eIF4Ai) ± 1 μM Navitoclax (BCL-xL/BCL-2i) ± 100 nM MRTX849 (KRASi) (mean ± SD, *n* = 3; *$p<0.05$, **$p<0.01$; one-way ANOVA and Tukey's post hoc test).

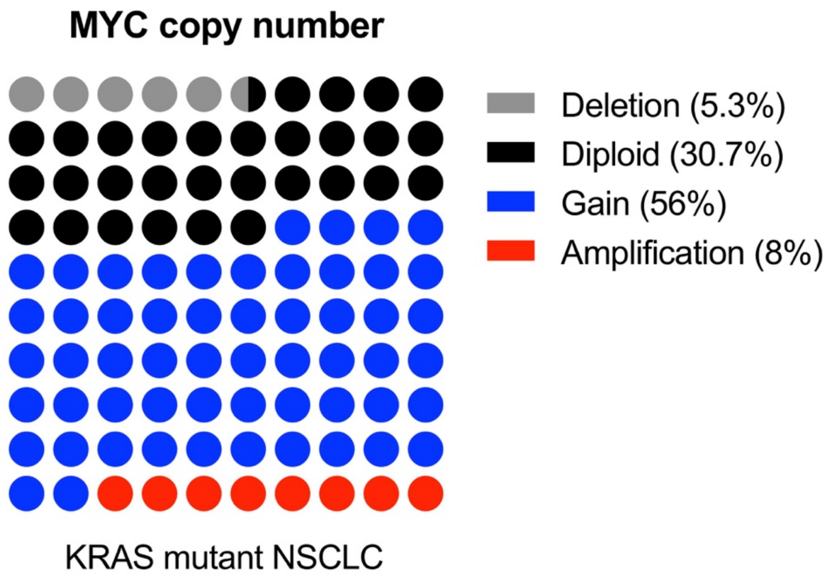
MYC copy number
Deletion (5.3%)
Diploid (30.7%)
Gain (56%)
Amplification (8%)
KRAS mutant NSCLC

Supplemental Figure 7. *KRAS*-mutant lung adenocarcinoma tumors frequently possess *MYC* copy number gain. *MYC* copy number for 75 *KRAS*-mutant NSCLC tumors is reported as deletion, diploid, copy number gain, or amplified.

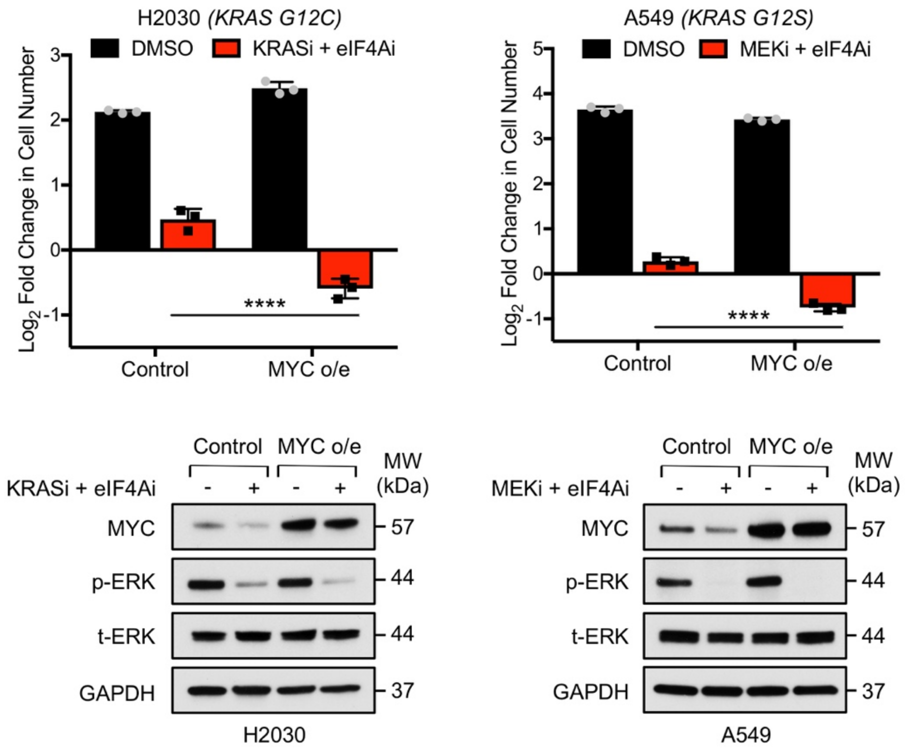

H2030 (KRAS G12C)
DMSO
KRASi + eIF4Ai
Log2 Fold Change in Cell Number
3
2
1
0
-1
****
Control
MYC o/e
A549 (KRAS G12S)
DMSO
MEKi + eIF4Ai
Log2 Fold Change in Cell Number
5
4
3
2
1
0
-1
****
Control
MYC o/e
Control
MYC o/e
MW (kDa)
KRASi + eIF4Ai
- + - +
MYC
57
p-ERK
44
t-ERK
44
GAPDH
37
H2030
Control
MYC o/e
MW (kDa)
MEKi + eIF4Ai
- + - +
MYC
57
p-ERK
44
t-ERK
44
GAPDH
37
A549

Supplemental Figure 8. MYC overexpression sensitizes resistant NSCLC cells to combined RAS pathway/eIF4A inhibitors. (Top) Bar graph depicting fold-change in cell number of resistant H2030 and A549 cells ectopically expressing control or *MYC* cDNAs treated for 72 hours with vehicle (DMSO) or combined 25 nM eFT226 (eIF4Ai) and either 100 nM MRTX849 (KRASi) or 50 nM trametinib (MEKi) (mean ± SD, n = 3; ****p<0.001; one-way ANOVA and Tukey's post hoc test). (Bottom) Immunoblots showing suppression of p-ERK and overexpression of MYC in response to specified treatments and *MYC* ectopic expression.
